# Digital Biomarker–Based Interventions: Systematic Review of Systematic Reviews

**DOI:** 10.2196/41042

**Published:** 2022-12-21

**Authors:** Hossein Motahari-Nezhad, Hana Al-Abdulkarim, Meriem Fgaier, Mohamed Mahdi Abid, Márta Péntek, László Gulácsi, Zsombor Zrubka

**Affiliations:** 1 Health Economics Research Center, University Research and Innovation Center, Óbuda University Budapest Hungary; 2 Doctoral School of Business and Management, Corvinus University of Budapest Budapest Hungary; 3 Doctoral School of Applied Informatics and Applied Mathematics, Óbuda University Budapest Hungary; 4 Drug Policy and Economic Center, National Guard Health Affairs, King Abdullah International Medical Research Center Riyadh Saudi Arabia; 5 Research Center of Epidemiology and Statistics, Université Sorbonne Paris Cité Paris France; 6 Corvinus Institute for Advanced Studies, Corvinus University of Budapest Budapest Hungary

**Keywords:** digital biomarker, digital health, digital devices, AMSTAR-2, GRADE, methodological quality, evidence synthesis, publication bias, imprecision, implantable, wearable

## Abstract

**Background:**

The introduction of new medical technologies such as sensors has accelerated the process of collecting patient data for relevant clinical decisions, which has led to the introduction of a new technology known as digital biomarkers.

**Objective:**

This study aims to assess the methodological quality and quality of evidence from meta-analyses of digital biomarker–based interventions.

**Methods:**

This study follows the PRISMA (Preferred Reporting Items for Systematic Reviews and Meta-Analyses) guideline for reporting systematic reviews, including original English publications of systematic reviews reporting meta-analyses of clinical outcomes (efficacy and safety endpoints) of digital biomarker–based interventions compared with alternative interventions without digital biomarkers. Imaging or other technologies that do not measure objective physiological or behavioral data were excluded from this study. A literature search of PubMed and the Cochrane Library was conducted, limited to 2019-2020. The quality of the methodology and evidence synthesis of the meta-analyses were assessed using AMSTAR-2 (A Measurement Tool to Assess Systematic Reviews 2) and GRADE (Grading of Recommendations, Assessment, Development, and Evaluations), respectively. This study was funded by the National Research, Development and Innovation Fund of Hungary.

**Results:**

A total of 25 studies with 91 reported outcomes were included in the final analysis; 1 (4%), 1 (4%), and 23 (92%) studies had high, low, and critically low methodologic quality, respectively. As many as 6 clinical outcomes (7%) had high-quality evidence and 80 outcomes (88%) had moderate-quality evidence; 5 outcomes (5%) were rated with a low level of certainty, mainly due to risk of bias (85/91, 93%), inconsistency (27/91, 30%), and imprecision (27/91, 30%). There is high-quality evidence of improvements in mortality, transplant risk, cardiac arrhythmia detection, and stroke incidence with cardiac devices, albeit with low reporting quality. High-quality reviews of pedometers reported moderate-quality evidence, including effects on physical activity and BMI. No reports with high-quality evidence and high methodological quality were found.

**Conclusions:**

Researchers in this field should consider the AMSTAR-2 criteria and GRADE to produce high-quality studies in the future. In addition, patients, clinicians, and policymakers are advised to consider the results of this study before making clinical decisions regarding digital biomarkers to be informed of the degree of certainty of the various interventions investigated in this study. The results of this study should be considered with its limitations, such as the narrow time frame.

**International Registered Report Identifier (IRRID):**

RR2-10.2196/28204

## Introduction

The introduction of new medical technologies such as sensors has accelerated the process of collecting patient data for relevant clinical decisions [1], which has led to the introduction of a new technology known as digital biomarkers (DBMs). “Digital biomarkers are objective, measurable, physiological, and behavioral parameters collected using wearable, portable, implantable, or digestible digital devices” [2]. DBMs can play an important role in daily clinical practice and clinical trials [3]. By providing timely and reliable disease-related information, DBMs can increase diagnostic accuracy, improve treatment decisions and help minimize clinical errors, and contribute to better patient outcomes [4-6]. Digital biomarkers can provide more reliable results than cross-sectional surveillance or prospective follow-up, allowing fewer patient visits [7]. Because of their growing importance in the health care value chain, the market of DBMs is expected to grow at a compound annual growth rate of 40.4% between 2019 and 2025, reaching a global revenue of US $5.64 billion by 2025 [8,9].

The rapid development of digital health technologies such as software [10], sensors [11], or robots [12,13] requires thorough examination and demonstration of their clinical effectiveness and economic benefits before they are widely deployed in publicly funded health systems. Assessing the value of digital health technologies is complex, with considerations beyond normal health economic analyses [14-18]. The evidence required for the value assessment of digital health technologies usually reflects their risk category ranging from basic consumer health monitoring to interventions impacting therapy or diagnosis. For high-risk technologies, it is essential to demonstrate the clinical benefit of randomized clinical trials conducted in a relevant health system or meta-analyses of randomized controlled trials [17,18].

In recent years, the clinical outcomes of DBMs have been extensively synthesized in systematic reviews and meta-analyses with inconsistent results, calling for a more systematic approach to evaluating the evidence concerning DBM interventions [19]. When interpreting systematic reviews, it is essential to appraise the quality of evidence and estimates of the effect size. Among the several methods for assessing the quality of evidence [20], the GRADE (Grading of Recommendations, Assessment, Development, and Evaluations) approach is used most commonly in systematic reviews, health technology assessments, and treatment guidelines [19]. GRADE classifies the quality of evidence into 4 categories from high to very poor [19]. However, poor reporting may limit the assessment of the quality of the evidence presented in systematic reviews. The AMSTAR-2 (A Measurement Tool to Assess Systematic Reviews 2) tool was developed to assess the methodological quality of systematic reviews [21].

Our goal, therefore, is to provide innovators and policymakers with actionable guidance on the level of evidence generation for DBMs, a rapidly growing area of medicine [2]. This systematic review of systematic reviews assesses the overall strength of evidence and methodological quality of systematic reviews that present a quantitative synthesis of the effects of digital biomarkers on health outcomes compared with interventions that do not include digital biomarkers. The AMSTAR-2 technique examines the methodological quality of studies, while GRADE assesses the overall quality of evidence based on digital biomarker technologies and reported outcomes.

## Methods

### Design and Protocol

This study follows the PRISMA (Preferred Reporting Items for Systematic Reviews and Meta-Analyses) guidelines for reporting systematic reviews (Multimedia Appendix 1) [22]. The protocol of the current systematic review was published in *JMIR Research Protocols* [23].

### Eligibility for Inclusion

DBMs are “objective, measurable, physiological, and behavioral parameters collected using wearable, portable, implantable, or digestible digital devices” [2]. In this research, we defined DBMs as either behavioral/physiological data or the digital devices used to collect these data. Wearable, implantable, or digestible medical devices or sensors that generate physiologic or behavioral data were considered digital biomarkers (eg, fitness trackers and defibrillators). Imaging or other technologies that do not measure physiological or behavioral data were excluded from this study. We interpret portable as “portable by patients or consumers”; therefore, portable devices operated by health care professionals (eg, digital stethoscopes) were excluded. We note that the definition of DBMs may overlap with sensor applications in the general population, such as citizen sensing [24]. In this search, we only considered systematic reviews that use digital devices deployed by clinicians or patients to collect clinical data in the context of treatment.

We included systematic reviews reporting meta-analyses of clinical outcomes of DBM-based interventions compared with alternative interventions without DBMs. In particular, we considered systematic reviews summarizing DBM-related evidence in a human population for any condition, age group, or sex. All interventions that use DBMs for any purpose related to diagnosing patients, monitoring outcomes, or influencing a therapeutic intervention were considered. There were no restrictions on comparators as long as the comparator arm did not involve using DBMs for the aforementioned purposes. Only meta-analyses of clinical outcomes were considered (ie, intended or unintended change in participants’ health status due to an intervention). Systematic reviews focused on the measurement properties, or other technical or utilization characteristics of DBMs that do not result in a change in participants’ health status were not eligible for this review. We considered full-text articles published in English in peer-reviewed journals between January 1, 2019, and December 31, 2020.

### Search Strategies

A literature search was conducted in PubMed and the Cochrane Library, with a time frame limited to 2019 and 2020. In addition, we checked the reference lists of systematic reviews potentially relevant to our research. The literature search used keywords related to “digital biomarkers” [2] in conjunction with The National Library of Medicine’s filter for “systematic reviews” [25] and the publication date. Multimedia Appendix 2 contains the complete search syntax.

### Screening and Selection

After removing duplicates, 2 reviewers (HM-N and MMA) independently screened titles and abstracts using 2 main criteria: (1) systematic reviews and (2) interventions that included DBMs. Reviewer calibration was performed after screening the titles/abstracts of the first 100 records using the following method. Both screening criteria were scored as either 1 (criterion not met) or 0 (criterion met or uncertain). Therefore, reviewers can evaluate each record with a score of 1, 2, 3, and 4, corresponding to the response patterns (0,0), (1,0), (0,1), and (1,1), respectively. Interrater agreement and κ statistics were calculated for scoring, and reviewers were retrained if worse than substantial agreement (κ=0.6) was observed [26]. A third reviewer (ZZ) made the decision in the case of nonmatching scores.

Full-text articles were assessed by 2 independent reviewers against all eligibility criteria: (1) English language; (2) human research; (3) publication date; (4) meta-analysis of clinical outcomes; (5) the intervention involved a DBM used for diagnosis, patient monitoring, or influencing therapy; (6) the comparator arm lacked a DBM for the same purposes. All 6 criteria had to be answered “yes” for inclusion. Discrepancies were resolved by the 2 reviewers. In case of disagreement, a third reviewer took a decision.

### Data Extraction and Quality Assessment

Data extraction and the assessments of methodological quality and the quality of evidence were performed by 2 independent researchers (HM-N, HA-A, or MF). Interrater agreement was assessed after completing data extraction from 20% of the included studies. Disagreements between reviewers were resolved by consensus, and a third reviewer (ZZ) resolved the remaining differences.

### Study-Level Variables

The following study-level variables were recorded: Year of publication; country of the first author; number of included studies in the qualitative/quantitative synthesis overall and separately for each outcome; study designs of the included studies (randomized controlled trial/nonrandomized controlled trial/cohort study/case-control study/cross-sectional study) [27]; population and its age range; the disease condition evaluated using the International Classification of Diseases 11th Revision (ICD-11) coding [28]; the number of included studies; intervention; type of intervention using the International Classification of Health Interventions (ICHI) coding [29]; comparator; type of comparator; the DBM; role of the DBM (diagnosis/patient monitoring/influencing intervention); body function quantified by the digital biomarker using the International Classification of Functioning, Disability and Health (ICF) coding [30]; and the list of synthesized outcomes.

### Outcome-Level Variables

We extracted the outcome measured, the total number of studies that examined that outcome, the total number of patients and the number receiving the intervention, the effect size and its 95% CI (upper and lower limits), and the type of effect size (eg, standardized mean difference/odds ratio/risk ratio).

### Assessment of the Methodological Quality of the Systematic Reviews

The methodological quality of the included systematic reviews was assessed using the AMSTAR-2 tool [21]. AMSTAR-2 is a recognized and reliable 16-item tool for evaluating the methodological quality of systematic reviews of health care treatments [21,31]. We performed a consistent assessment [32] using the AMSTAR-2 website and categorized the reporting quality of reviews accordingly as critically low, low, medium, and high [21].

### Assessing the Quality of the Evidence

We assessed the quality of evidence for each outcome using the GRADE system [19,33]. By default, GRADE classifies evidence from randomized controlled trials as high quality. However, this rating can be downgraded based on the assessment of the following 5 quality domains: (1) risk of bias [34], (2) inconsistency [35], (3) imprecision [36], (4) publication bias [37], and (5) indirectness [38]. Depending on the severity of the quality concerns, a downgrade of 0, 1, or 2 can be proposed for each domain.

We assessed the risk of bias according to the following criteria: if 75% or more than 75% of the included studies had a low risk of bias for a given outcome, no downgrade was applied. If less than 75% of the included studies had a low risk of bias or risk of bias was not reported, 1 downgrade was used [39].

Inconsistency was assessed by the reported heterogeneity for each outcome. If the *I*^2^ statistic was less than or equal to 75%, no downgrading was performed. If the *I*^2^ statistic was greater than 75%, 1 downgrade was assigned. If only a single study was included for the outcome, no downgrade was applied. If heterogeneity was not reported, a downgrade was applied [39].

Imprecision was assessed by evaluating the sample size [40]. The evidence was not downgraded if the pooled sample size exceeded 2000 [33]. We applied 1 downgrade if the pooled sample size was less than 200. Between a pooled sample size of 200 and 2000, we evaluated the optimal information size by power analysis using Stata version 16 (StataCorp LLC) as follows [33]: assuming a weak effect size [41], we calculated the sample size for a randomized controlled trial assuming a balanced sample, a power of 0.8, and a significance level of .05. One downgrade was applied when the calculated sample size was larger than the pooled sample size [33,40]. The following procedure was used for the small effect size: a Cohen *d* of 0.2 for continuous measures and 1.68 for the odds ratio. A weak effect size of 1.68 was also estimated for the risk ratio and hazard ratio, assuming a nonexposed prevalence of 0% [41,42].

The potential effect of publication bias on the effect size estimates was assessed for each outcome using the trim-and-fill method proposed by Duval and Tweedie [43]. Potentially missing studies were imputed, and the pooled effect size of the full data set was recalculated. If the imputation changed the conclusions of the analysis (eg, a significant effect size became no longer significant or the magnitude of effect size changed), we applied a downgrade due to publication bias [43]. According to the recommendations of the Cochrane Handbook [42], we assessed publication bias only in meta-analyses involving at least ten studies due to the limited power of risk of bias tests when applied on fewer studies.

When assessing indirectness for each outcome, we considered discrepancies between the included studies and the research question of the meta-analysis [44]. If the population, interventions, or comparators of the studies did not match the main objectives of the meta-analysis, a downgrade of 1 or 2 was considered, depending on the severity of this nonmatch, based on the consensus of the 2 independent investigators involved in data extraction.

The overall grading of the quality of evidence for each outcome was based on consensus, following the recommendation of Pollock et al [39]. The evidence was considered as high quality if further research was very unlikely to change our confidence in the estimate of effect (0 downgrades); moderate quality if further research was likely to have an important effect on our confidence in the estimate of effect and might change the estimate (1-2 downgrades); low quality if further research was very likely to have an important effect on our confidence in the estimate of effect and might change the estimate (3-4 downgrades); and very low quality if any estimate of the effect was very uncertain (5-6 downgrades) [19,39].

### Evidence Synthesis

Descriptive statistics including frequency and percentage were used to describe the characteristics of the studies using Stata version 16 and MS Excel 2016. The graphs were designed using R programming language 4.1.3 (R Core Team/R Foundation for Statistical Computing). In the designed graphs (Figures 2 and 3), the letters on the horizontal axis correspond to the interventions because the types of interventions were heterogeneous; for example, in 1 study, the intervention was a single digital device (such as an implantable cardiac defibrillator [ICD]), whereas in another, it was a combination of devices (such as Fitbit, Jawbone UP24, combined heart rate monitor, and accelerometer [Actiheart], wrist-worn accelerometer, FIT Core, Body Media, Fitbug Orb, Polar FA20 accelerometer). Given the diversity of populations and treatments studied, we tabulated the GRADE evidence summary for each DBM by type of intervention and outcome.

## Results

### Screening and Selection of Studies

**Figure 1:**
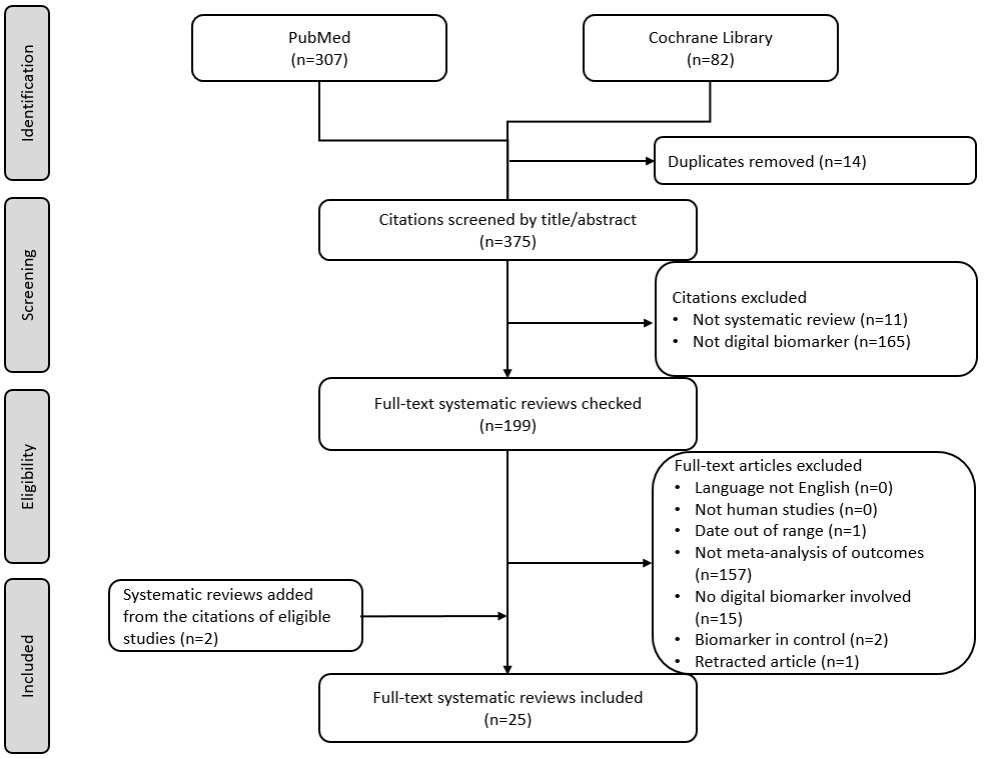
PRISMA (Preferred Reporting Items for Systematic Reviews and Meta-Analyses) diagram selecting/screening process.

Searches of the PubMed and Cochrane Library electronic databases yielded 307 and 82 documents, respectively, bringing the total number of studies found to 389. After removing duplicates (n=14), 375 studies were considered eligible for title/abstract screening. In the screening phase, we removed 176 studies, of which 11 were not systematic reviews and 165 did not involve DBMs (87 disagreements between reviewers during title/abstract screening; Cohen κ=0.54). During the screening phases of the titles/abstracts, “digital biomarker” was associated with 82 disagreements and “systematic review” with 5. Therefore, 199 studies were included in the full-text screening. In accordance with the eligibility criteria, 176 full-text papers were excluded (between-reviewers κ=0.76) for the following reasons: publication date outside the acceptable range (n=1), no meta-analysis of results (n=157), studies without DBMs (n=15), retraction (n=1) [45], and DBMs in the control group (n=2). The list of excluded studies with reasons are presented in Multimedia Appendix 3. In addition, when reviewing the reference lists of the final eligible studies, 2 more reviews met the inclusion criteria. Therefore, 25 systematic reviews were included in the final analysis (Figure 1).

### Characteristics of the Included Systematic Reviews

Most studies were published by authors from Australia (5/25, 20%) [46-50] followed by those from the United States (3/25, 12%) [51-53], Taiwan (3/25, 12%) [54-56], Canada (2/25, 8%) [57,58], Hong Kong (2/25, 8%) [59,60], and the United Kingdom of Great Britain and Northern Ireland (1/25, 4%) [61]. The other 9 reviews (36%) were published by researchers from Belgium [62], China [63], France [64], Greece [65], Japan [66], the Netherlands [67], Portugal [68], Saudi Arabia [69], and Thailand [70].

### Populations

Using ICD-11, most participants in the included systemic reviews were assigned to circulatory system diseases [47,48,51,56,57,60,62,65,67-70], followed by patients with endocrine, nutritional, or metabolic disorders [58,60,62,67] and respiratory system diseases [57,60-62,67]. Patients with nutritional disorders [47,60,62], diseases of the nervous system [47,60,67], and problems associated with health behaviors [47,59,67] were included in 3 reviews each. The other populations were classified in the presence of device, implants, or grafts [55,67]; diseases of the musculoskeletal system or connective tissue [47,64]; causes of health care–related harm or injury [52,53]; diseases of the urinary system [63]; injury or harm arising from surgical or medical care [55]; neoplasms [47]; and injury, poisoning, or certain other consequences of external causes [66]. In addition, 4 reviews examined nonclinical populations [46,49,50,54]. In some reviews, nonclinical cases such as healthy individuals [57,60], employees [57], and students [57] were included in addition to patients with clinical diseases that could not be categorized using the ICD-11 tool.

### Interventions

In accordance with the ICHI instrument, 14 diverse intervention categories were discovered, and the majority of digital biomarkers were used as interventions on physical activity behaviors (eg, Fitbit) [46-50,57-62,64,67], conversion of cardiac rhythm (eg, cardiac defibrillators) [51-53,63,68,69], cardiac electrophysiological monitoring (eg, iPhone-based rhythm monitoring device) [55,65,70], weight maintenance functions (eg, Garmin or Jawbone UP24) [49,54,57], and whole-body measurement (eg, wristbands and smartwatches) [50,54]. Other interventions identified were associated with cardiopulmonary resuscitation (eg, metronome with a siren) [56], assisting and leading exercise for exercise tolerance function (eg, GEx sensor of vital signs and smartphone) [48], body measurement of trunk (eg, wristbands, smartwatches) [54], pain (eg, accelerometer, pedometers) [64], test of functions (eg, YAMAX, Fitbit) [64], quality of life (eg, pedometers) [64], test of muscle endurance (eg, fitness trackers) [64], body measurement of lower limb (eg, accelerometer-based navigation system) [66], and test of maintaining body position (eg, accelerometer-based navigation system) [66].

### Outcomes

According to the ICF system, the vast majority of reported outcomes concerned physical activity (looking after one’s health; eg, moderate-to-vigorous physical activity, step counts) [46-50,58-62,64,67], followed by mortality (demographic change; eg, all-cause mortality, sudden cardiac death) [51-53,63,68-70], and heart functions (eg, return of spontaneous circulation, incidence of ventricular arrhythmia) [52,55,65,70]. A total of 11 studies also reported weight maintenance functions (eg, weight, BMI, and waist circumference) [49,54,57], health services, systems and policies (eg, quality of life and prevention) [55,63,64], maintaining one’s health (such as hospitalization and readmission rate) [51,52,69], and managing one’s own activity level (actions and behaviors to arrange the requirements in energy and time day-to-day procedures or duties; eg, sedentary behaviors) [46,57]. Because of the difference between sedentary behavior and physical activity, these 2 outcomes were considered different endpoints, as physical activity and sedentary behavior are measured differently and do not affect risks in the same way [71]. The other remaining reported outcomes were aerobic capacity [48], pain [64], fatigability [64], social security services, systems and policies (eg, disability) [64], body functions (eg, functional tests) [64], and mobility of joint functions (such as coronal femoral component alignment or coronal tibial component alignment) [66].

### Bodily Functions Quantified by Digital Biomarkers

The most commonly used physiological/behavioral data captured by digital biomarkers to modify participants’ health status were heart functions/rhythm [51-53,55,56,63,65,68,70] and physical activity (looking after one’s health) [46,47,50,57-60,64,67], followed by walking [46-49,59-62,64,67], weight maintenance functions [49,54,57], gait pattern functions [57], running [59], aerobic capacity [48], and involuntary movement reaction functions [66]. For further information regarding population, intervention, outcome, and digital biomarkers, see Multimedia Appendix 4.

### The Methodological Quality of Systematic Reviews

Most studies (23/25, 92%) [46-49,51-55,57-70] had critically low methodological quality according to the assessment using AMSTAR-2. The remaining studies also received high (1/25, 4%) [50] and low methodological quality (1/25, 4%) ratings [56]. The only study of high methodological quality was assigned to a review that investigated the effect of workplace pedometer interventions to increase physical activity [50]. Although all studies were able to meet criteria 3 (inclusion criteria), 9 (risk of bias assessment), and 11 (appropriate statistical methods) of AMSTAR-2, criteria 4 (comprehensive literature search), 7 (list of excluded studies), 10 (funding report), and 13 (account for risk of bias when reporting results) were met by only 2 [50,64], 2 [50,65], 2 [50,69], and 7 studies [46,50,54,56,58,60,66], respectively. Detailed information on the methodological quality of the studies for each criterion can be found in Multimedia Appendix 5.

### Quality of Evidence Synthesis Results

The 25 reviews included in the study comprised a total of 91 outcomes. Of the 91 outcomes, only 6 (7%) were rated as high-quality evidence, whereas 80 (88%) were rated as moderate-quality and 5 (5%) as low-quality evidence. The results showed that the effect of an ICD on all-cause mortality received high-quality evidence for ICDs implanted after and with continuous flow left ventricular assist devices. Furthermore, based on the analyses, we are highly confident about the impact of the ICD on the probability of transplantation, the detection rate of atrial arrhythmias, and the incidence of stroke. By contrast, some outcomes were found to have low-quality evidence, including the effect of wearable activity trackers on steps in chronic respiratory disease as well as on steps in overweight and sedentary older adults. A total of 2 meta-analyses that examined the effect of wearable activity trackers on moderate-to-vigorous physical activity were also rated as low-quality evidence. Concerning the criteria of GRADE, risk of bias was found in most outcomes (85/91, 93%), followed by inconsistency (27/91, 30%) and imprecision (27/91, 30%). Publication bias was detected in a small number of outcomes (2/91, 2%). By contrast, no indirectness was revealed in the outcomes. In addition, 67 outcomes (74%) were not examined for publication bias because the minimum number of included studies was insufficient; 3 outcomes (3%) were also not assessed for inconsistency because only 1 study was included. See Multimedia Appendix 6 for more details.

## Discussion

### Principal Findings

To our knowledge, this study is the first to analyze the methodological and evidence-based quality of systematic reviews providing meta-analyses of digital biomarker–based interventions’ effect on human populations’ health-related outcomes. A total of 25 systematic reviews evaluating the clinical impact of digital biomarkers on human health were included in our study, comprising a total of 91 outcomes. There were no reviews of high methodological quality on digital biomarker–based interventions with high quality of evidence. Most outcomes had moderate-quality evidence synthesis. All implantable cardiac devices and monitors had significant results with moderate-quality evidence and critically low methodological quality. Most activity trackers also had significant effects on steps and weight with moderate certainty of evidence and critically low methodological quality. By contrast, the evidence synthesis and methodological quality of activity trackers were rated moderate and critically low, respectively, for quality of life, pain, fatigue, and disability. Still, the results of the meta-analyses showed a nonsignificant effect of activity trackers on the aforementioned endpoints.

### The Methodological Quality of Systematic Reviews

The results of the methodological quality of the studies using the AMSTAR-2 tool showed that most studies had critically low methodological quality, mainly due to factor numbers 7 (excluded studies) and 10 (source of funding) of the AMSTAR-2 tool, leaving concerns about the unbiasedness of results and indicating the need for quality improvement. Researchers in this field need to follow the AMSTAR-2 guidelines and criteria to produce high-quality systematic reviews. The list of excluded studies and the rationale for deleting each study are critical parts of the AMSTAR-2 tool for assessment [21]. This limitation is included in the majority of some previously published systematic reviews in digital interventions for reducing behavioral risks [72], synchronous digital mental health systematic reviews [73], and interventions involving antibacterial envelopes to reduce cardiac implantable electronic device–related infections [74].

As listing excluded studies and the rationale for their deletion are critical components of the methodology of systematic reviews according to the AMSTAR-2 criteria [21], researchers are advised to provide excluded studies with rationale for their exclusion when conducting systematic reviews. In addition, the source of funding for the research included in the systematic reviews should be indicated. Most systematic reviews included in this study could not meet this criterion. The results of this study are consistent with those of many previous studies [72,73,75]. Prior studies on digital interventions for reducing behavioral risks [72] and systematic review of synchronous digital mental health reviews [73] also rated the methodological quality of most systematic reviews as critically low. By contrast, the methodological quality of most systematic reviews on digital health interventions on palliative care [75] and the use of eHealth with immunizations [76] was rated low and moderate, respectively.

### Quality of Evidence

Of the 91 outcomes assessed, only 6 had high-quality evidence, meaning that we can be highly confident that the actual effect is close to the estimated effect and that further studies are unlikely to change our confidence in the estimate of the effect [77]. Considering that a substantial proportion of digital biomarker–based outcomes had evidence of moderate quality, we have moderate confidence in the effect estimate. Although the actual effect is likely to be similar to the estimated effect, there is a possibility that it will be significantly different, and additional research is expected to have a significant impact on our confidence in the effect estimate and alter the estimate [77]. In addition, some outcomes were of low quality, suggesting that our confidence in the impact estimate is limited and that the actual effect may differ substantially from the impact estimate [77].

Most outcomes were downgraded mainly because of the risk of bias in the included studies. In addition, the analysis revealed that most of the included systematic reviews did not assess and discuss the impact of risk of bias on the measured outcomes. Therefore, clinical researchers in this field are advised first to determine the impact of risk of bias on their effect estimates and then discuss the likely impact of risk of bias on outcomes to produce high-quality results. High heterogeneity was another detrimental factor observed in nearly one-third of the outcomes. However, most of the included systematic reviews were able to meet AMSTAR criterion 14, investigated the sources of any heterogeneity in the results, and discussed this criterion’s impact on the review results. Researchers can study heterogeneity in several ways, such as by performing subgroup analyses or meta-regressions, using a fixed-effects or random-effects model [42], changing the statistical measure from risk difference to relative risk, and deleting studies [78]. Another critical factor in the deterioration of the quality of some outcomes was imprecision. Clinical researchers should consider the optimal information size for their measured outcomes using power calculations to obtain a high-quality effect estimate without imprecision.

Some previous studies also assessed the quality of evidence in some research areas. A study evaluating the quality of evidence of systematic reviews of acupuncture for stroke rehabilitation concluded that the quality of evidence for almost all outcomes was low, mainly because of inconsistency, imprecision, and risk of bias, respectively [79]. Another study that assessed the quality of meta-analyses of Chinese herbal preparations for the treatment of rheumatoid arthritis concluded that most outcomes (55%) were of low quality. In comparison, 25% and 20% were of moderate and very low quality, respectively, primarily because of the risk of bias and inconsistency [80]. Quality assessment of the evidence on the role of the dietary supplement curcumin in the treatment of ulcerative colitis yielded 10 moderate, 6 low, and 3 very low certainties of the evidence. The most deteriorating reasons were imprecision and publication bias [81]. The quality of evidence synthesis from meta-analyses on the effect of antibacterial envelopes in reducing infections associated with cardiac implantable electronic devices was found to be moderate in 60% of the outcomes in a recent paper, mostly due to the risk of bias and inconsistency [74].

**Figure 2:**
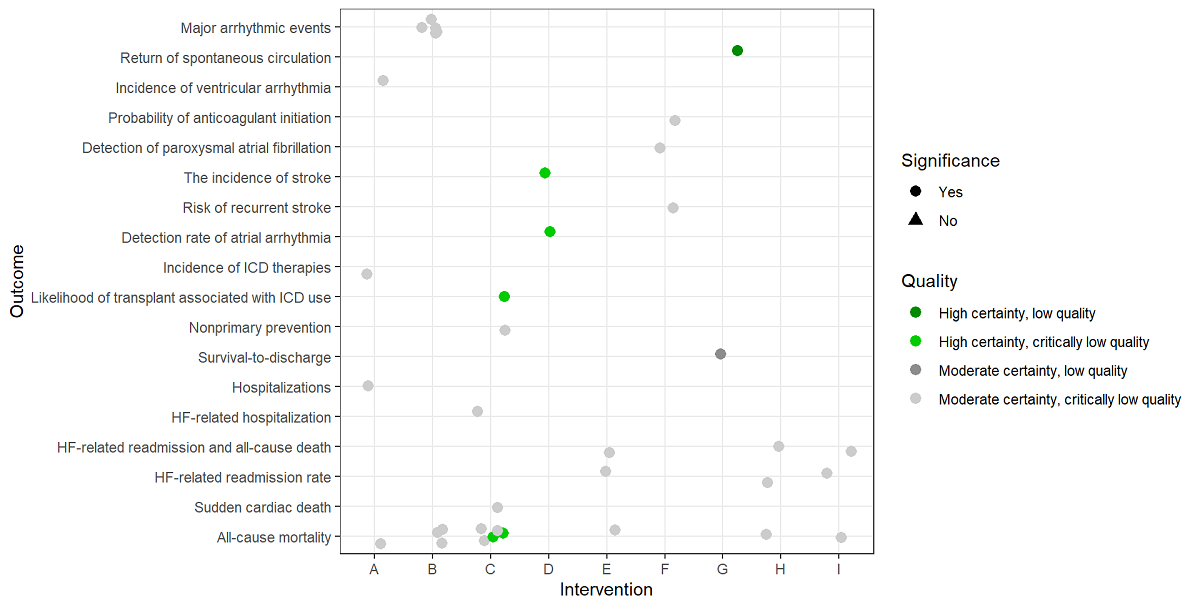
Cardiovascular-related interventions, outcomes, and methodological and evidence synthesis quality. HF: heart failure; ICD: implantable cardiac defibrillator. A: Cardiac resynchronization therapy, Implantable cardiac defibrillator [52], B: Fragmented QRS (fQRS) [70], C: Implantable cardiac defibrillator [53,63,68,69], D: Implantable cardiac defibrillator, iPhone-based rhythm monitoring device, pacemakers [55], E: Impedance devices [51], F: Implantable cardiac monitor, Holter-Electrocardiogram [65], G: Metronome with a siren, HeartStart-MRx, Zoll AED, Cardio First AngelTM [56], H: Pressure sensors [51], I: Pressure sensors and Impedance devices (Cardio MEMS, RVP sensor, Chronicle, ICD- OptiVol, InSync Sentry, lung impedance) [51].

As shown in Figure 2, all digital device interventions had significant effects on cardiac-related outcomes. According to the analyses results, we are highly confident that ICD has an impact on all-cause mortality (in 2 cases) and on the likelihood of transplantation. Moreover, we are highly confident about the impact of implantable and monitoring devices (ICD, iPhone-based rhythm monitoring device, and pacemakers) on the detection rate of atrial arrhythmias and stroke. Furthermore, the effect of some cardiac electronic devices (Metronome with a siren, HeartStart-MRx, Zoll AED, Cardio First Angel) on the return of spontaneous circulation created high-quality evidence but they come from studies with low and critically low methodological quality, which may raise some concerns about their results. The other interventions all have moderate-quality evidence synthesis, and we are moderately confident in the effect estimate. Furthermore, the actual effect is probably close to the effect estimate, but there is a possibility that it is substantially different. By contrast, these studies’ low and critically low methodological quality raise concerns about the validity of the effect estimates. More than 263,000 electronic cardiac devices have been implanted annually in Germany, France, and the United Kingdom [82]. Device therapy has become increasingly important in treating life-threatening heart disease [83]. As a result, patients, clinicians, and policymakers are advised to consider the results of this study when making medical decisions.

**Figure 3:**
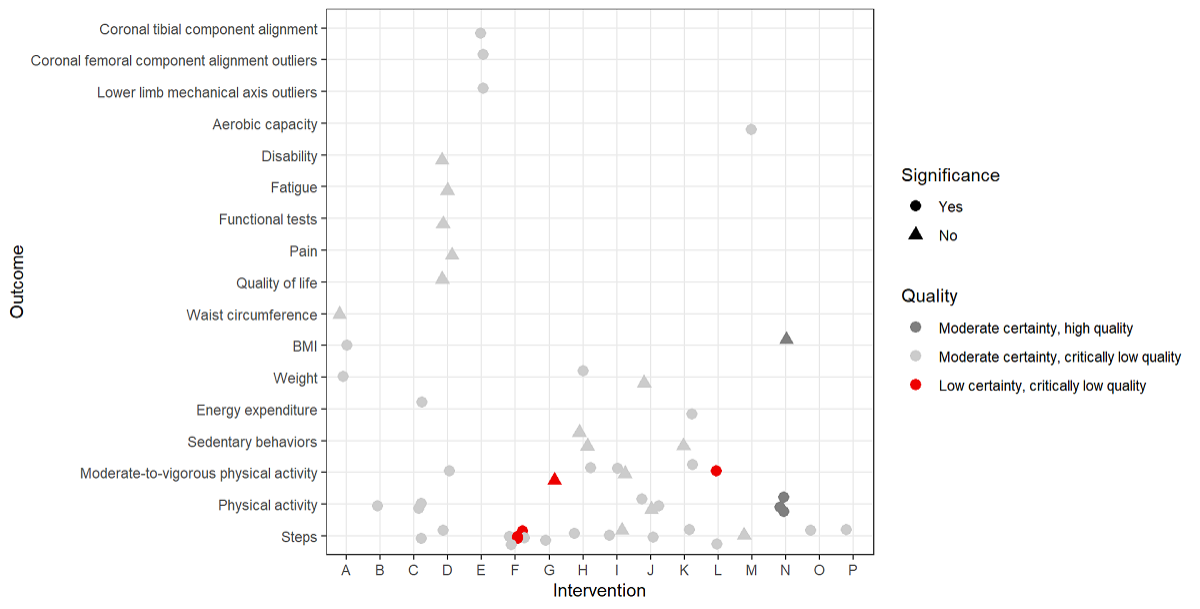
Activity trackers related to interventions, outcomes, and methodological and evidence synthesis quality. A: wristbands, smartwatches [54], B: Accelerometer, Dynaport MoveMonitor, Pedometer, Yamax Digi-walker CW700, ActivPal, ActiGraph, Personal Activity Monitor [67], C: Accelerometer, pedometer [60], D: Accelerometer, pedometers, Yamax, Fitbit [64], E: Accelerometer-based navigation system [66], F: wearable activity trackers (pedometer) [62], G: Activity monitor, portable tablet computers with touch screens, Fitbit, Jawbone UP24 wearable device, pedometer, accelerometer [59], H: Fitbit [57], I: Fitbit, Jawbone UP, Polar Active, Misfit Flash, Gruve Solution, LUMOback, BodyMedia Fit, SenseWear, ActiveLink, InBodyBand [47], J: Fitbit, Jawbone Up24, Combined heart rate monitor and accelerometer (Actiheart), Wrist-worn accelerometer, FIT Core, Body Media, Fitbug Orb, Polar FA20 accelerometer [49], K: Fitbit, Jawbone UP24, Gruve, LumoBack, Polar Active, Fitbug, Pebble+, Fitmeter, Personal Activity Monitor, Withings Pulse [46], L: Fitbit, Yorbody, AiperMotion [58], M: Garmin, Pedometer, Fitbit, Accelerometer, Yamax Digiwalker, Gex sensor of vital signs and smartphone [48], N: Pedometer [50], O: pedometer-based physical activity promotion [61], P: Pedometer physical activity promotion + pulmonary rehabilitation promotion [61].

Regarding the interventions with activity trackers (Figure 3), the vast majority had significant effects on human outcomes, whereas 16 outcomes were found to be ineffective in changing human health, including the effects of accelerometer, pedometers, YAMAX, Fitbit on disability, fatigue, functional tests, pain, and quality of life; the effects of activity monitor, portable tablet computers with touch screens, Fitbit, Jawbone UP24 wearable device, pedometer, and accelerometer on moderate-to-vigorous physical activity; the effect of Fitbit on sedentary behavior; the effect of Fitbit, Jawbone UP, Polar Active, Misfit Flash, Gruve Solution, LUMOback, BodyMedia FIT, SenseWear, ActiveLink, InBodyBand on moderate-to-vigorous physical activity (in 1 case) and on steps (in 1 case); the effect of Fitbit, Jawbone UP24, combined heart rate monitor and accelerometer (Actiheart), wrist-worn accelerometer, FIT Core, Body Media, Fitbug Orb, and Polar FA20 accelerometer on physical activity (in one case) and on weight; the effect of Fitbit, Jawbone UP24, Gruve, LUMOback, Polar Active, Fitbug, Pebble+, Fitmeter, personal activity monitor, Withings Pulse on sedentary behavior; the effect of Garmin, Pedometer, Fitbit, Accelerometer, YAMAX Digi-walker, GEx sensor of vital signs, and smartphone on steps; the effect of wristbands and smartwatches on waist circumference; and the effect of pedometer on BMI. Most of these had moderate-quality evidence synthesis from studies with critically low methodological quality. By contrast, our confidence in some effect estimates is limited, and the actual effects may differ substantially from the estimated effects, including the effect of pedometer on steps in chronic respiratory diseases, obesity, and in sedentary older adults; the effect of Fitbit, YorBody, AiperMotion on moderate-to-vigorous physical activity; and the effect of activity monitor, portable tablet computers with touch screens, Fitbit, Jawbone UP24 wearable device, pedometer, accelerometer on moderate-to-vigorous physical activity, which did not have even significant effect. Our distrust increases when we find that these results come from critically low methodological quality studies. Evidence of moderate quality, as shown in Figure 3, suggests that the use of pedometers may increase physical activity; these results are from a study with high methodological quality [50]. Other reported outcomes had moderate-quality evidence with critically low methodological quality. According to our analysis, and as shown in Figure 3, there is no high-quality evidence of the impact of activity trackers on human health behavior change.

### Strengths

Most systematic review studies performed in the field of digital biomarkers in recent years have mainly been conducted with a specific focus on 1 or more disease areas or technologies, such as the effects of wearable fitness trackers on motivation and physical activity or ICD troubleshooting in patients with left ventricular assist devices. To our knowledge, no comprehensive systematic review of systematic reviews of all types of digital biomarkers has been published in all populations and in all diseases. Therefore, our review aims to assess the quality of methods and evidence of systematic reviews without limiting it to a specific domain or technology, using validated tools and standard methods. As a result, the strength of evidence can be compared between different types of interventions, providing practical guidance for clinicians and policymakers. To our knowledge, this is the first comprehensive study to address the methodological and evidence-based quality of systematic reviews of digital biomarker–based interventions. To categorize populations, interventions, outcomes, and behavioral/physiological data in digital biomarkers, we used World Health Organization (WHO) standard tools such as ICD-11, ICHI, and ICF. In addition, the most validated assessment tools, AMSTAR-2 and GRADE, were used to assess the methodological quality and quality of evidence synthesis of the systematic reviews.

### Limitations

Despite the rigorous methodology, this study has some limitations, and readers are asked to consider the study’s results in light of its limitations. One of the study’s possible weaknesses is the short search duration (2019 and 2020). Only systematic reviews published in 2019 and 2020 were considered in this study according to the published protocol [23]. Because of the scope of the topic, we limited our assessment to a shorter period. However, given the new European Medical Devices Regulation (MDR) enacted in 2017 [84], we assumed this would be an exceptionally important period for evaluating clinical data collected before the regulations were implemented. While the 2-year period provides important insights into evidence syntheses published before MDR, longer periods would be needed to allow generalization of our findings.

As mentioned earlier, publication bias was assessed only in meta-analyses with at least ten studies. Of the 91 outcomes assessed, 67 included fewer than 10 studies, and we assessed publication bias in only 24 outcomes. In addition, the trim-and-fill approach, like any other method, may identify publication bias incorrectly in meta-analyses with a high degree of heterogeneity [85]. There were 2 outcomes where effect sizes were presented as a ratio of means. Thus, we interpreted the reported effect sizes as a mean difference to determine the optimal information size for assessing the imprecision. In 3 cases, the number of included studies in the meta-analyses was only 1. Therefore, an assessment of the quality of evidence was not possible for any of the GRADE criteria (risk of bias, publication bias, inconsistency, imprecision, and indirectness).

In our search, we operationalized the definition of digital biomarkers. However, we did not evaluate the sensitivity and specificity of our search filter for articles on digital biomarkers. Besides the broad terms we used in our search strategy, digital biomarkers can be identified using terms related to the technology or type of data collected [3]. However, creating a complete list of appropriate search terms for all available technologies was beyond the scope of this study and remains an unresolved research topic. Specific sensor applications in the general population may raise health concerns (eg, COVID-19 contact–tracking apps [86]) that were not considered in this research. As recommended in the relevant guidelines for the systematic review of systematic reviews, we searched only the PubMed and Cochrane databases for reviews, and we did not search the Database of Abstracts of Reviews of Effectiveness (DARE) [87]. The DARE was not used in this study because it does not contain reviews from 2015. In addition, our published protocol required us to search gray literature; however, due to the large number of outcomes from peer-reviewed sources, we did not search gray literature.

In our search based on the definition of digital biomarkers and the inclusion criteria, we may have overlooked papers on digital biomarkers that were not defined by terms without the key adjectives used in the definition, as described earlier. Examples include thermometers and continuous glucose monitors. Thus, because of the ambiguity of definitions in digital health, more comprehensive keyword collections in this area are needed, as these were concluded in a recently accepted scoping review of digital biomarkers [88] and an ISPOR (International Society for Pharmacoeconomics and Outcomes Research) report [89].

### Conclusion

In summary, we systematically reviewed the current evidence from systematic reviews on the use of digital biomarkers as interventions to change the health status of human populations. Overall, the 25 included current systematic reviews had critically low methodological quality, which may negatively affect the findings of the reported outcomes. In addition, most reported outcomes of interventions based on digital biomarkers had a moderate quality of evidence, implying that we have only moderate confidence in them. Only a small number of reported outcomes had high-quality evidence. Therefore, researchers in the field should consider the AMSTAR-2 criteria and GRADE to create future high-quality studies. Furthermore, patients, clinicians, and policymakers are advised to consider the results of this study before making clinical decisions relating to digital biomarkers.

## Appendix

Multimedia Appendix 1 PRISMA (Preferred Reporting Items for Systematic Reviews and Meta-Analyses) checklist.

Multimedia Appendix 2 Search strategies.

Multimedia Appendix 3 List of excluded studies.

Multimedia Appendix 4 Characteristics of the included studies.

Multimedia Appendix 5 Assessment of the methodological quality of reviews using the AMSTAR-2 (A Measurement Tool to Assess Systematic Reviews 2) tool.

Multimedia Appendix 6 Evidence summary and quality assessment by the GRADE (Grading of Recommendations, Assessment, Development, and Evaluations) tool.

## Abbreviations

AMSTAR-2: A Measurement Tool to Assess Systematic Reviews 2
DARE: Database of Abstracts of Reviews of Effectiveness
DBM: digital biomarker
GRADE: Grading of Recommendations, Assessment, Development, and Evaluations
HF: heart failure
ICD: implantable cardiac defibrillator
ICD-11: International Statistical Classification of Diseases and Related Health Problems
ICF: International Classification of Functioning, Disability and Health
ICHI: International Classification of Health Interventions
ISPOR: International Society for Pharmacoeconomics and Outcomes Research
MDR: Medical Devices Regulation
PRISMA: Preferred Reporting Items for Systematic Reviews and Meta-Analyses
WHO: World Health Organization
