# Supplementary material for: Digital Biomarker–Based Interventions: Systematic Review of Systematic Reviews
Source: J Med Internet Res. 2022 Dec 21;24(12):e41042. doi: 10.2196/41042 (PMC9813819; doi:10.2196/41042)
Supplement: Multimedia Appendix 2 [file jmir_v24i12e41042_app2.docx]

| Electronic database | Search strategy |
| --- | --- |
| PubMed | ("digital biomarker"[Title/Abstract] OR "digital biomarkers"[Title/Abstract] OR "portable"[Title/Abstract] OR "portables"[Title/Abstract] OR "wearable"[Title/Abstract] OR "wearables"[Title/Abstract] OR "implantable"[Title/Abstract] OR "implantables"[Title/Abstract] OR "digestible"[Title/Abstract] OR "digestibles"[Title/Abstract]) AND ((((("systematic review"[Title] OR "systematic literature review"[Title] OR "systematic scoping review"[Title] OR "systematic narrative review"[Title] OR "systematic qualitative review"[Title] OR "systematic evidence review"[Title] OR "systematic quantitative review"[Title] OR "systematic meta review"[Title] OR "systematic critical review"[Title] OR "systematic mixed studies review"[Title] OR "systematic mapping review"[Title] OR "systematic cochrane review"[Title] OR "systematic search and review"[Title] OR "systematic integrative review"[Title]) NOT "comment"[Publication Type]) NOT ("protocol"[Title] OR "protocols"[Title])) NOT "MEDLINE"[Filter]) OR ("cochrane database syst rev"[Journal] AND "review"[Publication Type]) OR "systematic review"[Publication Type]) AND 2019/01/01:2020/12/31[Date - Publication] |
| Cochrane Library | "digital biomarker" OR "digital biomarkers" OR portable OR portables OR wearable OR wearables OR implantable OR implantables OR digestible OR digestibles in Title Abstract Keyword - with Cochrane Library publication date Between Jan 2019 and Dec 2020, in Cochrane Reviews (Word variations have been searched) |

Search strategies.
