# Supplementary material for: Digital Biomarker–Based Interventions: Systematic Review of Systematic Reviews
Source: J Med Internet Res. 2022 Dec 21;24(12):e41042. doi: 10.2196/41042 (PMC9813819; doi:10.2196/41042)
Supplement: Multimedia Appendix 3 [file jmir_v24i12e41042_app3.docx]

List of excluded studies.

| No. | Title | Reason |
| --- | --- | --- |
| 1 | Wearable Health Technology to Quantify the Functional Impact of Peripheral Neuropathy on Mobility in Parkinson's Disease: A Systematic Review [1] | No meta-analysis of outcomes |
| 2 | Wearable Health Devices in Health Care: Narrative Systematic Review [2] | No meta-analysis of outcomes |
| 3 | Use of wearable biometric monitoring devices to measure outcomes in randomized clinical trials: a methodological systematic review [3] | No meta-analysis of outcomes |
| 4 | Biofeedback Systems for Gait Rehabilitation of Individuals with Lower-Limb Amputation: A Systematic Review [4] | No meta-analysis of outcomes |
| 5 | The Use of Activity Trackers in Interventions for Childhood Cancer Patients and Survivors: A Systematic Review [5] | No meta-analysis of outcomes |
| 6 | Predefined vs data-guided training prescription based on autonomic nervous system variation: A systematic review [6] | No meta-analysis of outcomes |
| 7 | Enabling Older Adults’ Health Self-Management through Self-Report and Visualization—A Systematic Literature Review [7] | No meta-analysis of outcomes |
| 8 | Effects of Motivational Interviewing and Wearable Fitness Trackers on Motivation and Physical Activity: A Systematic Review [8] | No meta-analysis of outcomes |
| 9 | Diagnostic Performance of Prehospital Point-of-Care Troponin Tests to Rule Out Acute Myocardial Infarction: A Systematic Review [9] | No meta-analysis of outcomes |
| 10 | Long-Term Weight Management Using Wearable Technology in Overweight and Obese Adults: Systematic Review [10] | No meta-analysis of outcomes |
| 11 | Sensing Technology to Facilitate Behavioral and Psychological Symptoms and to Monitor Treatment Response in People With Dementia. A Systematic Review [11] | No meta-analysis of outcomes |
| 12 | Association of Exposures to Seated Postures With Immediate Increases in Back Pain: A Systematic Review of Studies With Objectively Measured Sitting Time [12] | No meta-analysis of outcomes |
| 13 | Implantable cardiac monitors to detect atrial fibrillation after cryptogenic stroke: a systematic review and economic evaluation [13] | No meta-analysis of outcomes |
| 14 | Outcomes following implantable cardioverter– defibrillator generator replacement in adults: A systematic review [14] | No meta-analysis of outcomes |
| 15 | Can Mobile Technology Improve Weight Loss in Overweight Adults? A Systematic Review [15] | No meta-analysis of outcomes |
| 16 | Time trends in sudden cardiac death risk in heart failure patients with cardiac resynchronization therapy: a systematic review [16] | No meta-analysis of outcomes |
| 17 | The Role of Technology in Adherence to Physical Activity Programs in Patients with Chronic Diseases Experiencing Fatigue: a Systematic Review [17] | No meta-analysis of outcomes |
| 18 | Is There a Benefit to Patients Using Wearable Devices Such as Fitbit or Health Apps on Mobiles? A Systematic Review [18] | No meta-analysis of outcomes |
| 19 | Patient generated health data use in clinical practice: A systematic review [19] | No meta-analysis of outcomes |
| 20 | Frailty, Implantable Cardioverter Defibrillators, and Mortality: a Systematic Review [20] | No meta-analysis of outcomes |
| 21 | A Systematic Review of Electronic Health (eHealth) interventions to improve physical activity in patients with breast cancer [21] | No meta-analysis of outcomes |
| 22 | Mobile health interventions to promote physical activity and reduce sedentary behaviour in the workplace: A systematic review [22] | No meta-analysis of outcomes |
| 23 | Data management and wearables in older adults: A systematic review [23] | No meta-analysis of outcomes |
| 24 | Investigating the Use of an Electronic Activity Monitor System as a Component of Physical Activity and Weight-Loss Interventions in Nonclinical Populations: A Systematic Review [24] | No meta-analysis of outcomes |
| 25 | Validity and reliability of wearable inertial sensors in healthy adult walking: a systematic review and meta-analysis [25] | No meta-analysis of outcomes |
| 26 | Systematic Review of Randomized Controlled Trials of Exercise Interventions Using Digital Activity Trackers in Patients With Cancer [26] | No meta-analysis of outcomes |
| 27 | A Systematic Review of the Incidence of Arrhythmias in Hemodialysis Patients Undergoing Long-Term Monitoring With Implantable Loop Recorders [27] | No meta-analysis of outcomes |
| 28 | The Validity and Reliability of Wearable Microtechnology for Intermittent Team Sports: A Systematic Review [28] | No meta-analysis of outcomes |
| 29 | Assessment of Hypertension Using Clinical Electrocardiogram Features: A First-Ever Review [29] | No meta-analysis of outcomes |
| 30 | Comparison of Wearable Sensor to Traditional Methods in Functional Outcome Measures: A Systematic Review [30] | No meta-analysis of outcomes |
| 31 | Effects of Wearable Devices with Biofeedback on Biomechanical Performance of Running—A Systematic Review [31] | No meta-analysis of outcomes |
| 32 | The application of wearable smart sensors for monitoring the vital signs of patients in epidemics: a systematic literature review [32] | No meta-analysis of outcomes |
| 33 | The Use of Motor Cognitive Dual-Task Quantitative Assessment on Subjects with Mild Cognitive Impairment: A systematic Review [33] | No meta-analysis of outcomes |
| 34 | Implantable cardioverter-defibrillators in cardiac transplant recipients: A systematic review from the Electrophysiology Collaborative Consortium for Meta-analysis—ELECTRAM investigators [34] | No meta-analysis of outcomes |
| 35 | The Use of Wearables in Clinical Trials During Cancer Treatment: Systematic Review [35] | No meta-analysis of outcomes |
| 36 | Risk stratification using late gadolinium enhancement on cardiac magnetic resonance imaging in patients with hypertrophic cardiomyopathy: A systematic review and meta-analysis [36] | No meta-analysis of outcomes |
| 37 | Systematic review on the application of wearable inertial sensors to quantify everyday life motor activity in people with mobility impairments [37] | No meta-analysis of outcomes |
| 38 | Systematic review of context-aware digital behavior change interventions to improve health [38] | No meta-analysis of outcomes |
| 39 | Management of Systemic Fungal Infections in the Presence of a Cardiac Implantable Electronic Device: A Systematic Review [39] | No meta-analysis of outcomes |
| 40 | Mobile health applications for the detection of atrial fibrillation: a systematic review [40] | No meta-analysis of outcomes |
| 41 | Motion Capture Technology in Industrial Applications: A Systematic Review [41] | No meta-analysis of outcomes |
| 42 | Identification of Patient Perceptions That Can Affect the Uptake of Interventions Using Biometric Monitoring Devices: Systematic Review of Randomized Controlled Trials [42] | No meta-analysis of outcomes |
| 43 | Reliability and Validity of Commercially Available Wearable Devices for Measuring Steps, Energy Expenditure, and Heart Rate: Systematic Review [43] | No meta-analysis of outcomes |
| 44 | Wearable sensing devices for upper limbs: A systematic review [44] | No meta-analysis of outcomes |
| 45 | Electromagnetic interference effect of dental equipment on cardiac implantable electrical devices: A systematic review [45] | No meta-analysis of outcomes |
| 46 | The potential of functional near-infrared spectroscopy-based neurofeedback – a systematic review and recommendations for best practice [46] | No meta-analysis of outcomes |
| 47 | Systematic Review of Digital Phenotyping and Machine Learning in Psychosis Spectrum Illnesses [47] | No meta-analysis of outcomes |
| 48 | Better one or two? A systematic review of portable automated refractors [48] | No meta-analysis of outcomes |
| 49 | Current reporting of usability and impact of mHealth interventions for substance use disorder: A systematic review [49] | No meta-analysis of outcomes |
| 50 | Wearable Transdermal AlcoholMonitors: A Systematic Review of Detection Validity, and Relationship Between Transdermal and Breath Alcohol Concentration and Influencing Factors [50] | No meta-analysis of outcomes |
| 51 | Enabling Older Adults’ Health Self-Management through Self-Report and Visualization—A Systematic Literature Review [51] | No meta-analysis of outcomes |
| 52 | Effects of Motivational Interviewing and  Wearable Fitness Trackers on Motivation  and Physical Activity: A Systematic Review [52] | No meta-analysis of outcomes |
| 53 | Measuring Physical Activity Using Triaxial Wrist Worn Polar Activity Trackers:  A Systematic Review [53] | No meta-analysis of outcomes |
| 54 | Cost-Effectiveness of Extended Electrocardiogram  Monitoring for Atrial Fibrillation After Stroke [54] | No meta-analysis of outcomes |
| 55 | Mobile Health for Pediatric Weight Management: Systematic  Scoping Review [55] | No meta-analysis of outcomes |
| 56 | Current Evidence for Continuous Vital Signs Monitoring by  Wearable Wireless Devices in Hospitalized Adults: Systematic  Review [56] | No meta-analysis of outcomes |
| 57 | Objective measurement of sleep, heart rate, heart rate variability, and  physical activity in suicidality: A systematic review [57] | No meta-analysis of outcomes |
| 58 | Gait Kinematic Parameters in Parkinson’s  Disease: A Systematic Review [58] | No meta-analysis of outcomes |
| 59 | Wearable Solutions for Patients with Parkinson’s  Disease and Neurocognitive Disorder:  A Systematic Review [59] | No meta-analysis of outcomes |
| 60 | Wearable Inertial Sensors to Assess Gait during the  6-MinuteWalk Test: A Systematic Review [60] | No meta-analysis of outcomes |
| 61 | Technologies for Cognitive Training  and Cognitive Rehabilitation for  People With Mild Cognitive  Impairment and Dementia. A  Systematic Review [61] | No meta-analysis of outcomes |
| 62 | Subcutaneous implantable cardioverter-defibrillator troubleshooting in patients with  left ventricular assist devices: a case series and systematic review [62] | No meta-analysis of outcomes |
| 63 | Subcutaneous implantable cardioverter-defibrillator troubleshooting in patients with  left ventricular assist devices: a case series and systematic review [63] | No meta-analysis of outcomes |
| 64 | Evaluative Research of Technologies for Prehospital Communication  and Coordination: a Systematic Review [64] | No meta-analysis of outcomes |
| 65 | Clinical Applications of Mobile Health Wearable–Based Sleep  Monitoring: Systematic Review [65] | No meta-analysis of outcomes |
| 66 | The current use of wearable sensors to enhance safety and performance in breath-hold diving: A systematic review [66] | No meta-analysis of outcomes |
| 67 | A Systematic Review on the Use of Wearable Body  Sensors for Health Monitoring: A Qualitative Synthesis [67] | No meta-analysis of outcomes |
| 68 | Role of Wearable Accelerometer Devices in  Delirium Studies: A Systematic Review [68] | No meta-analysis of outcomes |
| 69 | Analysis of Relevant Features from  Photoplethysmographic Signals for Atrial  Fibrillation Classification [69] | No meta-analysis of outcomes |
| 70 | Perspectives of People Who Are Overweight and Obese on Using  Wearable Technology for Weight Management: Systematic Review [70] | No meta-analysis of outcomes |
| 71 | A systematic review of the accuracy of sleep wearable devices for estimating sleep  Onset [71] | No meta-analysis of outcomes |
| 72 | Are cost-effective technologies feasible to measure gait in older adults? A  systematic review of evidence-based literature [72] | No meta-analysis of outcomes |
| 73 | A Systematic Review of Evidence for  a Role of Rest-Activity Rhythms in  Dementia [73] | No meta-analysis of outcomes |
| 74 | Wearable systems for shoulder kinematics  assessment: a systematic review [74] | No meta-analysis of outcomes |
| 75 | Wearable Sensors for Monitoring of Cigarette  Smoking in Free-Living: A Systematic Review [75] | No meta-analysis of outcomes |
| 76 | Wearable Sensors for Monitoring of Cigarette  Smoking in Free-Living: A Systematic Review [76] | No meta-analysis of outcomes |
| 77 | A Systematic Review on the Value of Infrared Thermography  in the Early Detection of Periprosthetic Joint Infections [77] | No meta-analysis of outcomes |
| 78 | Analyzing the Use of Accelerometers as a Method of  Early Diagnosis of Alterations in Balance in Elderly  People: A Systematic Review [78] | No meta-analysis of outcomes |
| 79 | Current State of Digital Biomarker Technologies for Real-Life,  Home-Based Monitoring of Cognitive Function for Mild Cognitive  Impairment to Mild Alzheimer Disease and Implications for Clinical  Care: Systematic Review [79] | No meta-analysis of outcomes |
| 80 | A Systematic Review and Implementation of IoT-Based Pervasive  Sensor-Enabled Tracking System for Dementia Patients [80] | No meta-analysis of outcomes |
| 81 | A systematic review of feasibility studies promoting the use of  mobile technologies in clinical research [81] | No meta-analysis of outcomes |
| 82 | Quantification of the validity and reliability of sprint performance metrics  computed using inertial sensors: A systematic review [82] | No meta-analysis of outcomes |
| 83 | CanWe Rely on Mobile Devices and Other Gadgets to  Assess the Postural Balance of Healthy Individuals?  A Systematic Review [83] | No meta-analysis of outcomes |
| 84 | Digital biomarkers from geolocation data in bipolar disorder and schizophrenia: a systematic review [84] | No meta-analysis of outcomes |
| 85 | Pooled Analysis of the Efficacy and Safety of Video Capsule  Endoscopy in Patients with Implantable Cardiac Devices [85] | No meta-analysis of outcomes |
| 86 | The Cost-Effectiveness of Digital Health Interventions on the  Management of Cardiovascular Diseases: Systematic Review [86] | No meta-analysis of outcomes |
| 87 | A Systematic Review of Wearable Sensors and IoT-Based Monitoring  Applications for Older Adults – a Focus on Ageing Population  and Independent Living [87] | No meta-analysis of outcomes |
| 88 | The role of wearable devices and objective  gait analysis for the assessment and  monitoring of patients with lumbar spinal  stenosis: systematic review [88] | No meta-analysis of outcomes |
| 89 | Accuracy of mHealth Devices for Atrial Fibrillation Screening:  Systematic Review [89] | No meta-analysis of outcomes |
| 90 | The Role of the Cardioversion Defibrillator in  Post Myocardial Infarction Sudden Cardiac Death:  A Systematic Review of Clinical Trials and  Observational Studies [90] | No meta-analysis of outcomes |
| 91 | Assessing Eating Behaviour Using Upper Limb  Mounted Motion Sensors: A Systematic Review [91] | No meta-analysis of outcomes |
| 92 | Methodological considerations for kinematic  analysis of upper limbs in healthy and poststroke  adults Part II: a systematic review of motion  capture systems and kinematic metrics [92] | No meta-analysis of outcomes |
| 93 | Methodological considerations for kinematic  analysis of upper limbs in healthy and poststroke  adults Part II: a systematic review of motion  capture systems and kinematic metrics [93] | No meta-analysis of outcomes |
| 94 | Augmented Reality in Medicine: Systematic and Bibliographic  Review [94] | No meta-analysis of outcomes |
| 95 | Patient acceptability of wearable vital sign monitoring  technologies in the acute care setting: A systematic review [95] | No meta-analysis of outcomes |
| 96 | Exploring the Role ofWearable Technology in Sport  Kinematics and Kinetics: A Systematic Review [96] | No meta-analysis of outcomes |
| 97 | Current State and Future Directions of Technology-Based Ecological Momentary Assessment and Intervention for Major Depressive Disorder: A Systematic Review [97] | No meta-analysis of outcomes |
| 98 | Validity and Reliability of Wearable Sensors for Joint  Angle Estimation: A Systematic Review [98] | No meta-analysis of outcomes |
| 99 | Wearable activity trackers, accuracy, adoption, acceptance and health  impact: A systematic literature review [99] | No meta-analysis of outcomes |
| 100 | Reliability, Validity and Utility of Inertial Sensor Systems for Postural  Control Assessment in Sport Science and Medicine Applications:  A Systematic Review [100] | No meta-analysis of outcomes |
| 101 | The Key Factors in Physical Activity  Type Detection Using Real-Life Data:  A Systematic Review [101] | No meta-analysis of outcomes |
| 102 | Mobile Phone and Wearable Sensor-Based mHealth Approaches  for Psychiatric Disorders and Symptoms: Systematic Review [102] | No meta-analysis of outcomes |
| 103 | Assessing Gait in Parkinson’s Disease Using  Wearable Motion Sensors: A Systematic Review [103] | No meta-analysis of outcomes |
| 104 | The role of wearables in spinal posture  analysis: a systematic review [104] | No meta-analysis of outcomes |
| 105 | Post mortem pro life - Should we analyse the implantable devices after  death? A systematic review [105] | No meta-analysis of outcomes |
| 106 | The necessity of implantable cardioverter defibrillators in patients with  Kearns-Sayre syndrome - systematic review of the articles [106] | No meta-analysis of outcomes |
| 107 | Sleep Tracking: a Systematic Review of the Research Using  Commercially Available Technology [107] | No meta-analysis of outcomes |
| 108 | A Systematic Review of Technology-Driven  Methodologies for Estimation  of Energy Intake [108] | No meta-analysis of outcomes |
| 109 | Health-Related Quality of Life and Psychological Adjustment  of Children and Adolescents with Pacemakers and Implantable  Cardioverter Defibrillators: A Systematic Review [109] | No meta-analysis of outcomes |
| 110 | Impact of supportive therapy modalities on heart  rate variability in cancer patients – a systematic  review [110] | No meta-analysis of outcomes |
| 111 | Influence and safety of electronic apex locators in patients with  cardiovascular implantable electronic devices: a systematic review [111] | No meta-analysis of outcomes |
| 112 | Prognostic Value of Global Longitudinal  Strain in Hypertrophic Cardiomyopathy [112] | No meta-analysis of outcomes |
| 113 | A systematic review of effectiveness and economic evaluation  of Cardiohelp and portable devices for extracorporeal membrane  oxygenation (ECMO) [113] | No meta-analysis of outcomes |
| 114 | Electromagnetic interference in cardiac  electronic implants caused by novel electrical  appliances emitting electromagnetic fields in  the intermediate frequency range: a  systematic review [114] | No meta-analysis of outcomes |
| 115 | Automated Systems Based on Wearable Sensors  for the Management of Parkinson’s Disease at Home:  A Systematic Review [115] | No meta-analysis of outcomes |
| 116 | Detection of Near Falls Using Wearable Devices:  A Systematic Review [116] | No meta-analysis of outcomes |
| 117 | Digital contact tracing technologies in epidemics: a rapid review  (Review) [117] | No meta-analysis of outcomes |
| 118 | The effect of digital health technologies on managing symptoms across pediatric cancer continuum [118] | No meta-analysis of outcomes |
| 119 | Tracking health commodity inventory and notifying stock levels via mobile devices: a mixed methods systematic review [119] | No meta-analysis of outcomes |
| 120 | Barriers and facilitators to patient uptake and utilisation of digital interventions for the self-management of low back pain [120] | No meta-analysis of outcomes |
| 121 | Effectiveness, Acceptability, and Feasibility of Digital Health Interventions for LGBTIQ+ Young People [121] | No meta-analysis of outcomes |
| 122 | Digital and Mobile Technologies to Promote Physical Health Behavior Change and Provide Psychological Support for Patients Undergoing Elective Surgery [122] | No meta-analysis of outcomes |
| 123 | Instruments to assess self-efficacy among people with cardiovascular disease: A COSMIN systematic review [123] | No meta-analysis of outcomes |
| 124 | Asynchronous mHealth Interventions in Rheumatoid Arthritis: Systematic Scoping Review [124] | No meta-analysis of outcomes |
| 125 | A mixed methods systematic review of the effects of patient online self-diagnosing in the ‘smart-phone society’ on the healthcare professional-patient relationship and medical authority [125] | No meta-analysis of outcomes |
| 126 | mHealth for Integrated People-Centred Health Services in the Western Pacific: A Systematic Review [126] | No meta-analysis of outcomes |
| 127 | The efficacy of electronic health interventions targeting improved sleep for achieving prevention of weight gain in adolescents and young to middle-aged adults: A systematic review [127] | No meta-analysis of outcomes |
| 128 | Methodological Challenges in Randomized Controlled Trials on Smartphone-Based Treatment in Psychiatry: Systematic Review [128] | No meta-analysis of outcomes |
| 129 | Passive Sensing of Health Outcomes Through Smartphones: Systematic Review of Current Solutions and Possible Limitations [129] | No meta-analysis of outcomes |
| 130 | Mobile technology in health (mHealth) and antenatal care-Searching for apps and available solutions: A systematic review [130] | No meta-analysis of outcomes |
| 131 | Hearables: New Perspectives and Pitfalls of In-Ear Devices for Physiological Monitoring. A Scoping Review [131] | No meta-analysis of outcomes |
| 132 | Current clinical utilisation of wearable motion sensors for the assessment of outcome following knee arthroplasty: a scoping review [132] | No meta-analysis of outcomes |
| 133 | Using wearables to assess bradykinesia and rigidity in patients with Parkinson's disease: a focused, narrative review of the literature [133] | No meta-analysis of outcomes |
| 134 | Real-time monitoring technology in single-case experimental design research: Opportunities and challenges [134] | No meta-analysis of outcomes |
| 135 | Current Device Therapies for Sudden Cardiac Death Prevention - the ICD, Subcutaneous ICD and Wearable ICD [135] | No meta-analysis of outcomes |
| 136 | Mobile technologies to support healthcare provider to healthcare provider communication and management of care [136] | No meta-analysis of outcomes |
| 137 | Health workers’ perceptions and experiences of using mHealth technologies to deliver primary healthcare services: a qualitative evidence synthesis [137] | No meta-analysis of outcomes |
| 138 | A systematic review of portable electronic technology for health education in resource-limited settings [138] | No meta-analysis of outcomes |
| 139 | Cardiac resynchronization therapy: a comprehensive review [139] | No meta-analysis of outcomes |
| 140 | Value of gait analysis for measuring disease severity using inertial sensors in patients with multiple sclerosis: protocol for a systematic review and meta-analysis [140] | No meta-analysis of outcomes |
| 141 | Acceptance and Use of Innovative Assistive Technologies among People with Cognitive Impairment and Their Caregivers: A Systematic Review [141] | No meta-analysis of outcomes |
| 142 | Remote Patient Monitoring: A Systematic Review [142] | No meta-analysis of outcomes |
| 143 | Engaging Children and Young People in Digital Mental Health Interventions: Systematic Review of Modes of Delivery, Facilitators, and Barriers [143] | No meta-analysis of outcomes |
| 144 | Wearable Devices for Ambulatory Cardiac Monitoring: JACC State-of-the-Art Review [144] | No meta-analysis of outcomes |
| 145 | Digital health technology and mobile devices for the management of diabetes mellitus: state of the art [145] | No meta-analysis of outcomes |
| 146 | Mobile Apps for Health Behavior Change in Physical Activity, Diet, Drug and Alcohol Use, and Mental Health: Systematic Review [146] | No meta-analysis of outcomes |
| 147 | Digital Interventions to Support Adolescents and Young Adults With Cancer: Systematic Review [147] | No meta-analysis of outcomes |
| 148 | Mobile health applications for people with dementia: a systematic review and synthesis of qualitative studies [148] | No meta-analysis of outcomes |
| 149 | A systematic review of the behaviour change techniques and digital features in technology-driven type 2 diabetes prevention interventions [149] | No meta-analysis of outcomes |
| 150 | Effects of Mobile Health Including Wearable Activity Trackers to Increase Physical Activity Outcomes Among Healthy Children and Adolescents: Systematic Review [150] | No meta-analysis of outcomes |
| 151 | Digital exercise interventions for improving measures of central obesity: a systematic review [151] | No meta-analysis of outcomes |
| 152 | Digital interventions for people living with non-communicable diseases in India: A systematic review of intervention studies and recommendations for future research and development [152] | No meta-analysis of outcomes |
| 153 | A systematic review on clinical implication of continuous glucose monitoring in diabetes management [153] | No meta-analysis of outcomes |
| 154 | Duration of Implantable Cardiac Monitoring and Detection of Atrial Fibrillation in Ischemic Stroke Patients: A Systematic Review and Meta-Analysis [154] | No meta-analysis of outcomes |
| 155 | How well do activity monitors estimate energy expenditure? A systematic review and meta-analysis of the validity of current technologies [155] | No meta-analysis of outcomes |
| 156 | Timing of device reimplantation and reinfection rates following cardiac  implantable electronic device infection: a systematic review and meta-analysis [156] | No meta-analysis of outcomes |
| 157 | Validity of Wrist-Worn photoplethysmography devices to measure heart rate: A systematic review and meta-analysis [157] | No meta-analysis of outcomes |
| 158 | Baseline fragmented QRS is associated with increased all-cause mortality in heart failure with reduced ejection fraction: A systematic review and meta-analysis [158] | Publication date |
| 159 | The use of wearable devices in chronic disease management to enhance adherence and improve telehealth outcomes: A systematic review and meta-analysis [159] | Digital biomarker in control group |
| 160 | Inertial sensors versus standard systems in gait analysis: A systematic review and meta-analysis [160] | Digital biomarker in control group |
| 161 | The Effectiveness of Wearable Upper Limb Assistive Devices in Degenerative Neuromuscular Diseases: A Systematic Review and Meta-Analysis [161] | No digital biomarker involved |
| 162 | The effectiveness of mHealth interventions on postpartum depression: A systematic review and meta-analysis [162] | No digital biomarker involved |
| 163 | The prognostic value of late gadolinium enhancement in myocarditis and clinically suspected myocarditis: systematic review and meta-analysis [163] | No digital biomarker involved |
| 164 | Accuracy of Portable Face-Scanning Devices for Obtaining Three-Dimensional Face Models: A Systematic Review and Meta-Analysis [164] | No digital biomarker involved |
| 165 | Radiofrequency catheter ablation of ventricular tachycardia in ischemic heart disease in light of current practice: a systematic review and meta-analysis of randomized controlled trials [165] | No digital biomarker involved |
| 166 | Prolonging the flush-lock interval of totally implantable venous access ports in patients with cancer: A systematic review and meta-analysis [166] | No digital biomarker involved |
| 167 | Meta-analysis of medical management versus catheter ablation for atrial fibrillation [167] | No digital biomarker involved |
| 168 | Catheter Ablation of Atrial Fibrillation in Patients With Heart Failure: A Meta-analysis of Randomized Controlled Trials [168] | No digital biomarker involved |
| 169 | Meta-analysis comparing outcomes of catheter ablation for ventricular arrhythmia in ischemic versus nonischemic cardiomyopathy [169] | No digital biomarker involved |
| 170 | Effect of assisted walking-movement in patients with genetic and acquired neuromuscular disorders with the motorised Innowalk device: an international case study meta-analysis [170] | No digital biomarker involved |
| 171 | Left atrial structure and function predictors of recurrent fibrillation after catheter ablation: a systematic review and meta-analysis [171] | No digital biomarker involved |
| 172 | Meta-analysis Comparing Fluorescence Imaging with Radioisotope and Blue Dye-Guided Sentinel Node Identification for Breast Cancer Surgery [172] | No digital biomarker involved |
| 173 | Efficacy of glaucoma drainage devices in uveitic glaucoma and a meta-analysis of the literature [173] | No digital biomarker involved |
| 174 | The effectiveness and safety of acupuncture for depression: An overview of meta-analyses [174] | No digital biomarker involved |
| 175 | Electro-acupuncture vs. sham electro-acupuncture for chronic severe functional constipation: A systematic review and meta-analysis [175] | No digital biomarker involved |
| 176 | Accelerometer- and Pedometer-Based Physical Activity Interventions among Adults with Cardiometabolic Conditions: A Systematic Review and Meta-analysis [176] | Retracted article |

**References**

1. Corrà MF, Warmerdam E, Vila-Chã N, Maetzler W, Maia L. Wearable health technology to quantify the functional impact of peripheral neuropathy on mobility in Parkinson’s disease: A systematic review. Sensors (Switzerland) 2020;20(22):1–34. PMID:33228056

2. Lu L, Zhang J, Xie Y, Gao F, Xu S, Wu X, Ye Z. Wearable health devices in health care: Narrative systematic review. JMIR mHealth uHealth 2020;8(11). PMID:33164904

3. Graña Possamai C, Ravaud P, Ghosn L, Tran VT. Use of wearable biometric monitoring devices to measure outcomes in randomized clinical trials: a methodological systematic review. BMC Med BMC Medicine; 2020;18(1):1–11. PMID:33153462

4. Escamilla-Nunez R, Michelini A, Andrysek J. Biofeedback systems for gait rehabilitation of individuals with lower-limb amputation: A systematic review. Sensors (Switzerland) 2020;20(6). PMID:32183338

5. Ha L, Mizrahi D, Wakefield CE, Cohn RJ, Simar D, Signorelli C. The Use of Activity Trackers in Interventions for Childhood Cancer Patients and Survivors: A Systematic Review. J Adolesc Young Adult Oncol 2021;10(1):1–14. PMID:32897805

6. Düking P, Zinner C, Reed JL, Holmberg HC, Sperlich B. Predefined vs data-guided training prescription based on autonomic nervous system variation: A systematic review. Scand J Med Sci Sport 2020;30(12):2291–2304. PMID:32785959

7. Cajamarca G, Herskovic V, Rossel PO. Enabling older adults’ health self-management through self-report and visualization—a systematic literature review†. Sensors (Switzerland) 2020;20(15):1–16. PMID:32759801

8. Nuss K, Moore K, Nelson T, Li K. Effects of Motivational Interviewing and Wearable Fitness Trackers on Motivation and Physical Activity: A Systematic Review. Am J Heal Promot 2021;35(2):226–235. PMID:32662277

9. Alghamdi A, Alotaibi A, Alharbi M, Reynard C, Body R. Diagnostic Performance of Prehospital Point-of-Care Troponin Tests to Rule out Acute Myocardial Infarction: A Systematic Review. Prehosp Disaster Med 2020;35(5):567–573. PMID:32641173

10. Fawcett E, van Velthoven MH, Meinert E. Long-term weight management using wearable technology in overweight and obese adults: Systematic review. JMIR mHealth uHealth 2020;8(3):1–10. PMID:32154788

11. Husebo BS, Heintz HL, Berge LI, Owoyemi P, Rahman AT, Vahia I V. Sensing technology to facilitate behavioral and psychological symptoms and to monitor treatment response in people with dementia: A systematic review. Front Pharmacol 2020;10(February):1–13. [doi: 10.3389/fphar.2019.01699]

12. De Carvalho DE, de Luca K, Funabashi M, Breen A, Wong AYL, Johansson MS, Ferreira ML, Swab M, Neil Kawchuk G, Adams J, Hartvigsen J. Association of Exposures to Seated Postures With Immediate Increases in Back Pain: A Systematic Review of Studies With Objectively Measured Sitting Time. J Manipulative Physiol Ther [Internet] Elsevier Ltd; 2020;43(1):1–12. PMID:32081511

13. Edwards SJ, Wakefield V, Jhita T, Kew K, Cain P, Marceniuk G. Implantable cardiac monitors to detect atrial fibrillation after cryptogenic stroke: A systematic review and economic evaluation. Health Technol Assess (Rockv) [Internet] 2020;24(5):v–184. PMID:31944175

14. McCarthy KJ, Locke AH, Coletti M, Young D, Merchant FM, Kramer DB. Outcomes following implantable cardioverter–defibrillator generator replacement in adults: A systematic review. Hear Rhythm [Internet] Heart Rhythm Society; 2020;17(6):1036–1042. PMID:31931173

15. Wang E, Abrahamson K, Liu PJ, Ahmed A. Can Mobile Technology Improve Weight Loss in Overweight Adults? A Systematic Review. West J Nurs Res 2020;42(9):747–759. PMID:31762402

16. Barra S, Providência R, Narayanan K, Boveda S, Duehmke R, Garcia R, Leyva F, Roger V, Jouven X, Agarwal S, Levy WC, Marijon E. Time trends in sudden cardiac death risk in heart failure patients with cardiac resynchronization therapy: A systematic review. Eur Heart J 2020;41(21):1976–1986. PMID:31750896

17. Albergoni A, Hettinga FJ, La Torre A, Bonato M, Sartor F. The Role of Technology in Adherence to Physical Activity Programs in Patients with Chronic Diseases Experiencing Fatigue: a Systematic Review. Sport Med - Open Sports Medicine - Open; 2019;5(1). [doi: 10.1186/s40798-019-0214-z]

18. Jo A, Coronel BD, Coakes CE, Mainous AG. Is There a Benefit to Patients Using Wearable Devices Such as Fitbit or Health Apps on Mobiles? A Systematic Review. Am J Med [Internet] Elsevier Inc.; 2019;132(12):1394-1400.e1. PMID:31302077

19. Demiris G, Iribarren SJ, Sward K, Lee S, Yang R. Patient generated health data use in clinical practice: A systematic review. Nurs Outlook [Internet] 2019 Jul;67(4):311–330. [doi: 10.1016/j.outlook.2019.04.005]

20. Chen MY, Orkaby AR, Rosenberg MA, Driver JA. Frailty, Implantable Cardioverter Defibrillators, and Mortality: a Systematic Review. J Gen Intern Med Journal of General Internal Medicine; 2019;34(10):2224–2231. PMID:31264082

21. Dorri S, Asadi F, Olfatbakhsh A, Kazemi A. A Systematic Review of Electronic Health (eHealth) interventions to improve physical activity in patients with breast cancer. Breast Cancer [Internet] Springer Japan; 2020;27(1):25–46. PMID:31187411

22. Buckingham SA, Williams AJ, Morrissey K, Price L, Harrison J. Mobile health interventions to promote physical activity and reduce sedentary behaviour in the workplace: A systematic review. Digit Heal 2019;5:1–50. [doi: 10.1177/2055207619839883]

23. Alharbi M, Straiton N, Smith S, Neubeck L, Gallagher R. Data management and wearables in older adults: A systematic review. Maturitas [Internet] Elsevier; 2019;124(February):100–110. PMID:30910279

24. Sypes EE, Newton G, Lewis ZH. Investigating the use of an electronic activity monitor system as a component of physical activity and weight-loss interventions in nonclinical populations: A systematic review. J Phys Act Heal 2019;16(4):294–302. PMID:30849927

25. Kobsar D, Charlton JM, Tse CTF, Esculier JF, Graffos A, Krowchuk NM, Thatcher D, Hunt MA. Validity and reliability of wearable inertial sensors in healthy adult walking: A systematic review and meta-analysis. J Neuroeng Rehabil Journal of NeuroEngineering and Rehabilitation; 2020;17(1):1–21. PMID:32393301

26. Schaffer K, Panneerselvam N, Loh KP, Herrmann R, Kleckner IR, Dunne RF, Lin P, Heckler CE, Gerbino N, Bruckner LB, Storozynsky E, Ky B, Baran A, Mohile G, Mustian KM, Fung C. HHS Public Access. 2020;17(1):57–63. [doi: 10.6004/jnccn.2018.7082.Systematic]

27. Roberts PR, Stromberg K, Johnson LC, Wiles BM, Mavrakanas TA, Charytan DM. A Systematic Review of the Incidence of Arrhythmias in Hemodialysis Patients Undergoing Long-Term Monitoring With Implantable Loop Recorders. Kidney Int Reports [Internet] Elsevier Inc; 2021;6(1):56–65. [doi: 10.1016/j.ekir.2020.10.020]

28. Crang ZL, Duthie G, Cole MH, Weakley J, Hewitt A, Johnston RD. The Validity and Reliability of Wearable Microtechnology for Intermittent Team Sports: A Systematic Review. Sport Med [Internet] Springer International Publishing; 2021;51(3):549–565. [doi: 10.1007/s40279-020-01399-1]

29. Bird K, Chan G, Lu H, Greeff H, Allen J, Abbott D, Menon C, Lovell NH, Howard N, Chan WS, Fletcher RR, Alian A, Ward R, Elgendi M. Assessment of Hypertension Using Clinical Electrocardiogram Features: A First-Ever Review. Front Med 2020;7(December):1–17. PMID:33344473

30. Follis S, Chen Z, Mishra S, Howe CL, Toosizadeh N, Dohm M. Comparison of wearable sensor to traditional methods in functional outcome measures: A systematic review. J Orthop Res 2021;39(10):2093–2102. PMID:33300119

31. Giraldo-Pedroza A, Lee WC-C, Lam W-K, Coman R, Alici G. Effects of Wearable Devices with Biofeedback on Biomechanical Performance of Running—A Systematic Review. Sensors [Internet] 2020 Nov 19;20(22):6637. [doi: 10.3390/s20226637]

32. Mohammadzadeh N, Gholamzadeh M, Saeedi S, Rezayi S. The application of wearable smart sensors for monitoring the vital signs of patients in epidemics: a systematic literature review. J Ambient Intell Humaniz Comput [Internet] Springer Berlin Heidelberg; 2020;(0123456789). [doi: 10.1007/s12652-020-02656-x]

33. Mancioppi G, Fiorini L, Rovini E, Cavallo F. The use of Motor and Cognitive Dual-Task quantitative assessment on subjects with mild cognitive impairment: A systematic review. Mech Ageing Dev [Internet] Elsevier Ireland Ltd; 2021;193:111393. PMID:33188785

34. Garg J, Shah K, Turagam MK, Tzou W, Gopinathannair R, Natale A, Lakkireddy D. Implantable cardioverter-defibrillators in cardiac transplant recipients: A systematic review from the Electrophysiology Collaborative Consortium for Meta-analysis—ELECTRAM investigators. PACE - Pacing Clin Electrophysiol 2020;43(12):1529–1537. PMID:33180346

35. Beauchamp UL, Pappot H, Holländer-Mieritz C. The use of wearables in clinical trials during cancer treatment: Systematic review. JMIR mHealth uHealth 2020;8(11):1–15. PMID:33174852

36. Kamp NJ, Chery G, Kosinski AS, Desai MY, Wazni O, Schmidler GS, Patel M, Lopes RD, Morin DP, Al-Khatib SM. Risk stratification using late gadolinium enhancement on cardiac magnetic resonance imaging in patients with hypertrophic cardiomyopathy: A systematic review and meta-analysis. Prog Cardiovasc Dis [Internet] Elsevier Inc; 2021;66:10–16. PMID:33171204

37. Rast FM, Labruyère R. Systematic review on the application of wearable inertial sensors to quantify everyday life motor activity in people with mobility impairments. J Neuroeng Rehabil [Internet] BioMed Central; 2020;17(1). PMID:33148315

38. Thomas Craig KJ, Morgan LC, Chen CH, Michie S, Fusco N, Snowdon JL, Scheufele E, Gagliardi T, Sill S. Systematic review of context-aware digital behavior change interventions to improve health. Transl Behav Med 2021;11(5):1037–1048. PMID:33085767

39. Baman JR, Medhekar AN, Jain SK, Knight BP, Harrison LH, Smith B, Saba S. Management of systemic fungal infections in the presence of a cardiac implantable electronic device: A systematic review. PACE - Pacing Clin Electrophysiol 2021;44(1):159–166. PMID:33052591

40. Lopez Perales CR, Van Spall HGC, Maeda S, Jimenez A, Laţcu DG, Milman A, Kirakoya-Samadoulougou F, Mamas MA, Muser D, Casado Arroyo R. Mobile health applications for the detection of atrial fibrillation: A systematic review. Europace 2021;23(1):11–28. PMID:33043358

41. Menolotto M, Komaris DS, Tedesco S, O’flynn B, Walsh M. Motion capture technology in industrial applications: A systematic review. Sensors (Switzerland) 2020;20(19):1–25. PMID:33028042

42. Perlmutter A, Benchoufi M, Ravaud P, Tran VT. Identification of patient perceptions that can affect the uptake of interventions using biometric monitoring devices: Systematic review of randomized controlled trials. J Med Internet Res 2020;22(9). PMID:32915153

43. Fuller D, Colwell E, Low J, Orychock K, Ann Tobin M, Simango B, Buote R, van Heerden D, Luan H, Cullen K, Slade L, Taylor NGA. Reliability and Validity of Commercially Available Wearable Devices for Measuring Steps, Energy Expenditure, and Heart Rate: Systematic Review. JMIR mHealth uHealth 2020;8(9):1–23. PMID:32897239

44. Dong M, Fang B, Li J, Sun F, Liu H. Wearable sensing devices for upper limbs: A systematic review. Proc Inst Mech Eng Part H J Eng Med 2021;235(1):117–130. PMID:32885713

45. Niu Y, Chen Y, Li W, Xie R, Deng X. Electromagnetic interference effect of dental equipment on cardiac implantable electrical devices: A systematic review. PACE - Pacing Clin Electrophysiol 2020;43(12):1588–1598. PMID:32852847

46. Kohl SH, Mehler DMA, Lührs M, Thibault RT, Konrad K, Sorger B. The Potential of Functional Near-Infrared Spectroscopy-Based Neurofeedback—A Systematic Review and Recommendations for Best Practice. Front Neurosci 2020;14. [doi: 10.3389/fnins.2020.00594]

47. Benoit J, Onyeaka H, Keshavan M, Torous J. Systematic Review of Digital Phenotyping and Machine Learning in Psychosis Spectrum Illnesses. Harv Rev Psychiatry 2020;28(5):296–304. PMID:32796192

48. Samanta A, Shetty A, Nelson PC. Better one or two? A systematic review of portable automated refractors. J Telemed Telecare 2020; [doi: 10.1177/1357633X20940140]

49. Carreiro S, Newcomb M, Leach R, Ostrowski S, Boudreaux ED, Amante D. Current reporting of usability and impact of mHealth interventions for substance use disorder: A systematic review. Drug Alcohol Depend [Internet] Elsevier; 2020;215(August):108201. PMID:32777691

50. van Egmond K, Wright CJC, Livingston M, Kuntsche E. Wearable Transdermal Alcohol Monitors: A Systematic Review of Detection Validity, and Relationship Between Transdermal and Breath Alcohol Concentration and Influencing Factors. Alcohol Clin Exp Res 2020;44(10):1918–1932. PMID:32767791

51. Villarreal V, Berbey-Alvarez A. Evaluation of mhealth applications related to cardiovascular diseases: A systematic review. Acta Inform Medica 2020;28(2):130–137. [doi: 10.5455/aim.2020.28.130-137]

52. Nuss K, Moore K, Nelson T, Li K. Effects of Motivational Interviewing and Wearable Fitness Trackers on Motivation and Physical Activity: A Systematic Review. Am J Heal Promot 2020;1–10. [doi: 10.1177/0890117120939030]

53. Henriksen A, Johansson J, Hartvigsen G, Grimsgaard S, Hopstock L. Measuring physical activity using triaxial wrist worn polar activity trackers: A systematic review. Int J Exerc Sci 2020;13(4):438–454. PMID:32509122

54. Chew DS, Rennert-May E, Spackman E, Mark DB, Exner D V. Cost-effectiveness of extended electrocardiogram monitoring for atrial fibrillation after stroke a systematic review. Stroke 2020;51(7):2244–2248. PMID:32498661

55. Tully L, Burls A, Sorensen J, El-Moslemany R, O’Malley G. Mobile health for pediatric weight management: Systematic scoping review. JMIR mHealth uHealth 2020;8(6):1–15. PMID:32490849

56. Leenen JPL, Leerentveld C, van Dijk JD, van Westreenen HL, Schoonhoven L, Patijn GA. Current evidence for continuous vital signs monitoring by wearable wireless devices in hospitalized adults: Systematic review. J Med Internet Res 2020;22(6). PMID:32469323

57. Kang GE, Patriquin MA, Nguyen H, Oh H, Rufino KA, Storch EA, Schanzer B, Mathew SJ, Salas R, Najafi B. Objective measurement of sleep, heart rate, heart rate variability, and physical activity in suicidality: A systematic review. J Affect Disord [Internet] Elsevier B.V.; 2020;273(January):318–327. PMID:32421619

58. Bouça-Machado R, Jalles C, Guerreiro D, Pona-Ferreira F, Branco Di, Guerreiro T, Matias R, Ferreira JJ. Gait Kinematic Parameters in Parkinson’s Disease: A Systematic Review. J Parkinsons Dis 2020;10(3):843–853. PMID:32417796

59. Channa A, Popescu N, Ciobanu V. Wearable solutions for patients with parkinson’s disease and neurocognitive disorder: A systematic review. Sensors (Switzerland) 2020;20(9). PMID:32397516

60. Storm FA, Cesareo A, Reni G, Biffi E. Wearable inertial sensors to assess gait during the 6-minute walk test: A systematic review. Sensors (Switzerland) 2020;20(9). PMID:32384806

61. Irazoki E, Contreras-Somoza LM, Toribio-Guzmán JM, Jenaro-Río C, Van Der Roest H, Franco-Martín MA. Technologies for cognitive training and cognitive rehabilitation for people with mild cognitive impairment and dementia. A systematic review. Front Psychol 2020;11(April). PMID:32373018

62. Black-Maier E, Lewis RK, Barnett AS, Pokorney SD, Sun AY, Koontz JI, Daubert JP, Piccini JP. Subcutaneous implantable cardioverter-defibrillator troubleshooting in patients with a left ventricular assist device: A case series and systematic review. Hear Rhythm [Internet] Heart Rhythm Society; 2020;17(9):1536–1544. PMID:32304733

63. Morgan C, Rolinski M, McNaney R, Jones B, Rochester L, Maetzler W, Craddock I, Whone AL. Systematic Review Looking at the Use of Technology to Measure Free-Living Symptom and Activity Outcomes in Parkinson’s Disease in the Home or a Home-like Environment. J Parkinsons Dis 2020;10(2):429–454. PMID:32250314

64. Zhang Z, Brazil J, Ozkaynak M, Desanto K. Evaluative Research of Technologies for Prehospital Communication and Coordination: a Systematic Review. J Med Syst 2020;44(5). PMID:32246206

65. Guillodo E, Lemey C, Simonnet M, Walter M, Baca-García E, Masetti V, Moga S, Larsen M, Ropars J, Berrouiguet S. Clinical applications of mobile health wearable–based sleep monitoring: Systematic review. JMIR mHealth uHealth 2020;8(4):1–10. [doi: 10.2196/10733]

66. Vinetti G, Lopomo NF, Taboni A, Fagoni N, Ferretti G. The current use of wearable sensors to enhance safety and performance in breath-hold diving: A systematic review. Diving Hyperb Med 2020;50(1):54–65. PMID:32187619

67. Kristoffersson A, Lindén M. A systematic review on the use of wearable body sensors for health monitoring: A qualitative synthesis. Sensors (Switzerland) 2020;20(5). PMID:32182907

68. Davoudi A, Manini TM, Bihorac A, Rashidi P. Role of Wearable Accelerometer Devices in Delirium Studies. Crit Care Explor 2019;1(9):e0027. [doi: 10.1097/cce.0000000000000027]

69. Millán CA, Girón NA, Lopez DM. Analysis of relevant features from photoplethysmographic signals for atrial fibrillation classification. Int J Environ Res Public Health 2020;17(2). PMID:31941071

70. Hu R, van Velthoven MH, Meinert E. Perspectives of people who are overweight and obese on using wearable technology for weight management: Systematic review. JMIR mHealth uHealth 2020;8(1). PMID:31929104

71. Scott H, Lack L, Lovato N. A systematic review of the accuracy of sleep wearable devices for estimating sleep onset. Sleep Med Rev [Internet] Elsevier Ltd; 2020;49:101227. PMID:31901524

72. Zhong R, Rau PLP. Are cost-effective technologies feasible to measure gait in older adults? A systematic review of evidence-based literature. Arch Gerontol Geriatr [Internet] Elsevier; 2020;87(October 2019):103970. PMID:31743825

73. Smagula SF, Gujral S, Capps CS, Krafty RT. A systematic review of evidence for a role of rest-activity rhythms in dementia. Front Psychiatry 2019;10(OCT):1–7. [doi: 10.3389/fpsyt.2019.00778]

74. Carnevale A, Longo UG, Schena E, Massaroni C, Lo Presti D, Berton A, Candela V, Denaro V. 肩关节运动评估的可穿戴系统:系统综述. BMC Musculoskelet Disord BMC Musculoskeletal Disorders; 2019;20(1).

75. Imtiaz MH, Ramos-Garcia RI, Wattal S, Tiffany S, Sazonov E. Wearable sensors for monitoring of cigarette smoking in free-living: A systematic review. Sensors (Switzerland) 2019;19(21). PMID:31661856

76. Rozin Kleiner AF, Belgamo A, Pagnussat AS, Costa e Silva A de A, Sforza C, Rocha NACF. Wearable sensors, cerebral palsy and gait assessment in everyday environments: Is it a reality? - A systematic review. Funct Neurol 2019;34(2):85–91. PMID:31556388

77. Scheidt S, Rüwald J, Schildberg FA, Mahlein AK, Seuser A, Wirtz DC, Jacobs C. A Systematic Review on the Value of Infrared Thermography in the Early Detection of Periprosthetic Joint Infections. Z Orthop Unfall 2020;158(4):397–405. PMID:31525794

78. Leirós-Rodríguez R, García-Soidán JL, Romo-Pérez V. Analyzing the use of accelerometers as a method of early diagnosis of alterations in balance in elderly people: A systematic review. Sensors (Switzerland) 2019;19(18). PMID:31505828

79. Piau A, Wild K, Mattek N, Kaye J. Current state of digital biomarker technologies for real-life, home-based monitoring of cognitive function for mild cognitive impairment to mild Alzheimer disease and implications for clinical care: Systematic review. J Med Internet Res 2019;21(8). PMID:31471958

80. Ray PP, Dash D, De D. A Systematic Review and Implementation of IoT-Based Pervasive Sensor-Enabled Tracking System for Dementia Patients. J Med Syst Journal of Medical Systems; 2019;43(9). PMID:31317281

81. Bakker JP, Goldsack JC, Clarke M, Coravos A, Geoghegan C, Godfrey A, Heasley MG, Karlin DR, Manta C, Peterson B, Ramirez E, Sheth N, Bruno A, Bullis E, Wareham K, Zimmerman N, Forrest A, Wood WA. A systematic review of feasibility studies promoting the use of mobile technologies in clinical research. npj Digit Med [Internet] Springer US; 2019;2(1). [doi: 10.1038/s41746-019-0125-x]

82. Macadam P, Cronin J, Neville J, Diewald S. Quantification of the validity and reliability of sprint performance metrics computed using inertial sensors: A systematic review. Gait Posture [Internet] Elsevier; 2019;73(July):26–38. PMID:31299501

83. Pinho AS, Salazar AP, Hennig EM, Spessato BC, Domingo A, Pagnussat AS. Can we rely on mobile devices and other gadgets to assess the postural balance of healthy individuals? A systematic review. Sensors (Switzerland) 2019;19(13). PMID:31284455

84. Fraccaro P, Beukenhorst A, Sperrin M, Harper S, Palmier-Claus J, Lewis S, Van Der Veer SN, Peek N. Digital biomarkers from geolocation data in bipolar disorder and schizophrenia: A systematic review. J Am Med Informatics Assoc 2019;26(11):1412–1420. PMID:31260049

85. Tabet R, Nassani N, Karam B, Shammaa Y, Akhrass P, Deeb L. Pooled Analysis of the Efficacy and Safety of Video Capsule Endoscopy in Patients with Implantable Cardiac Devices. Can J Gastroenterol Hepatol Hindawi; 2019;2019:2–7. PMID:31236386

86. Jiang X, Ming WK, You JHS. The cost-effectiveness of digital health interventions on the management of cardiovascular diseases: Systematic review. J Med Internet Res 2019;21(6):1–11. PMID:31210136

87. Baig MM, Afifi S, GholamHosseini H, Mirza F. A Systematic Review of Wearable Sensors and IoT-Based Monitoring Applications for Older Adults – a Focus on Ageing Population and Independent Living. J Med Syst 2019;43(8):10916. PMID:31203472

88. Chakravorty A, Mobbs RJ, Anderson DB, Rooke K, Phan K, Yoong N, Maharaj M, Choy WJ. The role of wearable devices and objective gait analysis for the assessment and monitoring of patients with lumbar spinal stenosis: Systematic review. BMC Musculoskelet Disord BMC Musculoskeletal Disorders; 2019;20(1):1–9. PMID:31202276

89. Giebel GD, Gissel C. Accuracy of mhealth devices for atrial fibrillation screening: Systematic review. JMIR mHealth uHealth 2019;7(6):1–13. PMID:31199337

90. Maqsood MH, Rubab K. The Role of the Cardioversion Defibrillator in Post Myocardial Infarction Sudden Cardiac Death: A Systematic Review of Clinical Trials and Observational Studies. Cureus 2019;11(3). [doi: 10.7759/cureus.4314]

91. Heydarian H, Adam M, Burrows T, Collins C, Rollo ME. Assessing eating behaviour using upper limb mounted motion sensors: A systematic review. Nutrients 2019;11(5). PMID:31137677

92. Mesquita IA, Fonseca PFP da, Pinheiro ARV, Velhote Correia MFP, Silva CIC da. Methodological considerations for kinematic analysis of upper limbs in healthy and poststroke adults Part II: a systematic review of motion capture systems and kinematic metrics. Top Stroke Rehabil [Internet] Taylor & Francis; 2019;26(6):464–472. PMID:31064281

93. Yenikomshian M, Jarvis J, Patton C, Yee C, Mortimer R, Birnbaum H, Topash M. Cardiac arrhythmia detection outcomes among patients monitored with the Zio patch system: a systematic literature review. Curr Med Res Opin [Internet] Taylor & Francis; 2019;35(10):1659–1670. PMID:31045463

94. Eckert M, Volmerg JS, Friedrich CM. Augmented reality in medicine: Systematic and bibliographic review. JMIR mHealth uHealth 2019;7(4). [doi: 10.2196/10967]

95. Sprogis SK, Currey J, Considine J. Patient acceptability of wearable vital sign monitoring technologies in the acute care setting: A systematic review. J Clin Nurs 2019;28(15–16):2732–2744. PMID:31017338

96. Adesida Y, Papi E, McGregor AH. Exploring the role of wearable technology in sport kinematics and kinetics: A systematic review. Sensors (Switzerland) 2019;19(7). PMID:30987014

97. Colombo D, Fernández-Álvarez J, Patané A, Semonella M, Kwiatkowska M, García-Palacios A, Cipresso P, Riva G, Botella C. Current state and future directions of technology-based ecological momentary assessment and intervention for major depressive disorder: A systematic review. J Clin Med 2019;8(4). [doi: 10.3390/jcm8040465]

98. Poitras I, Dupuis F, Bielmann M, Campeau-Lecours A, Mercier C, Bouyer LJ, Roy JS. Validity and reliability ofwearable sensors for joint angle estimation: A systematic review. Sensors (Switzerland) 2019;19(7):1–17. PMID:30935116

99. Shin G, Jarrahi MH, Fei Y, Karami A, Gafinowitz N, Byun A, Lu X. Wearable activity trackers, accuracy, adoption, acceptance and health impact: A systematic literature review. J Biomed Inform [Internet] Elsevier; 2019;93(March):103153. PMID:30910623

100. Johnston W, O’Reilly M, Argent R, Caulfield B. Reliability, Validity and Utility of Inertial Sensor Systems for Postural Control Assessment in Sport Science and Medicine Applications: A Systematic Review [Internet]. Sport Med. Springer International Publishing; 2019. PMID:30903440ISBN:0123456789

101. Allahbakhshi H, Hinrichs T, Huang H, Weibel R. The key factors in physical activity type detection using real-life data: A systematic review. Front Physiol 2019;10(FEB):1–20. [doi: 10.3389/fphys.2019.00075]

102. Seppälä J, De Vita I, Jämsä T, Miettunen J, Isohanni M, Rubinstein K, Feldman Y, Grasa E, Corripio I, Berdun J, D’Amico E, Bulgheroni M. Mobile phone and wearable sensor-based mhealth approaches for psychiatric disorders and symptoms: Systematic review. JMIR Ment Heal 2019;6(2):1–14. PMID:30785404

103. Brognara L, Palumbo P, Grimm B, Palmerini L. Assessing Gait in Parkinson’s Disease Using Wearable Motion Sensors: A Systematic Review. Diseases 2019;7(1):18. [doi: 10.3390/diseases7010018]

104. Simpson L, Maharaj MM, Mobbs RJ. The role of wearables in spinal posture analysis: A systematic review. BMC Musculoskelet Disord BMC Musculoskeletal Disorders; 2019;20(1):1–14. PMID:30736775

105. Dyrbuś M, Tajstra M, Gąsior M. Post mortem pro life - Should we analyse the implantable devices after death? A systematic review. Int J Cardiol 2019;280:89–94. PMID:30658926

106. Imamura T, Sumitomo N, Muraji S, Mori H, Osada Y, Oyanagi T, Kojima T, Yoshiba S, Kobayashi T, Ono K. The necessity of implantable cardioverter defibrillators in patients with Kearns-Sayre syndrome - systematic review of the articles -. Int J Cardiol [Internet] Elsevier B.V.; 2019;279:105–111. PMID:30642644

107. Robbins R, Seixas A, Walton Masters L, Chanko N, Diaby F, Vieira D, Jean-Louis G. Sleep Tracking: a Systematic Review of the Research Using Commercially Available Technology. Curr Sleep Med Reports Current Sleep Medicine Reports; 2019;5(3):156–163. [doi: 10.1007/s40675-019-00150-1]

108. Doulah A, Mccrory MA, Higgins JA, Sazonov E. A Systematic Review of Technology-Driven Methodologies for Estimation of Energy Intake. IEEE Access IEEE; 2019;7:49653–49668. [doi: 10.1109/ACCESS.2019.2910308]

109. Pyngottu A, Werner H, Lehmann P, Balmer C. Health-Related Quality of Life and Psychological Adjustment of Children and Adolescents with Pacemakers and Implantable Cardioverter Defibrillators: A Systematic Review. Pediatr Cardiol [Internet] Springer US; 2019;40(1):1–16. PMID:30539238

110. Palma S, Keilani M, Hasenoehrl T, Crevenna R. Impact of supportive therapy modalities on heart rate variability in cancer patients–a systematic review. Disabil Rehabil [Internet] Taylor & Francis; 2020;42(1):36–43. PMID:30512975

111. AlRahabi MK, Ghabbani HM. Influence and safety of electronic apex locators in patients with cardiovascular implantable electronic devices: a systematic review. Libyan J Med [Internet] Taylor & Francis; 2019;14(1). PMID:30458679

112. Tower-Rader A, Mohananey D, To A, Lever HM, Popovic ZB, Desai MY. Prognostic Value of Global Longitudinal Strain in Hypertrophic Cardiomyopathy: A Systematic Review of Existing Literature. JACC Cardiovasc Imaging 2019;12(10):1930–1942. PMID:30219395

113. Mahboub-Ahari A, Heidari F, Sadeghi-Ghyassi F, Asadi M. A systematic review of effectiveness and economic evaluation of Cardiohelp and portable devices for extracorporeal membrane oxygenation (ECMO). J Artif Organs [Internet] Springer Japan; 2019;22(1):6–13. PMID:30187234

114. Driessen S, Napp A, Schmiedchen K, Kraus T, Stunder D. Electromagnetic interference in cardiac electronic implants caused by novel electrical appliances emitting electromagnetic fields in the intermediate frequency range: A systematic review. Europace 2019;21(2):219–229. PMID:29992289

115. Rovini E, Maremmani C, Cavallo F. Automated Systems Based on Wearable Sensors for the Management of Parkinson’s Disease at Home: A Systematic Review. Telemed e-Health 2019;25(3):167–183. PMID:29969384

116. Pang I, Okubo Y, Sturnieks D, Lord SR, Brodie MA. Detection of Near Falls Using Wearable Devices: A Systematic Review. J Geriatr Phys Ther 2019;42(1):48–56. PMID:29384813

117. Anglemyer A. Digital contact tracing technologies in epiDemics: A rapid review. Saudi Med J 2020;41(9):1028. PMID:33502000

118. Cheng L, Duan M, Mao X, Ge Y, Wang Y, Huang H. The effect of digital health technologies on managing symptoms across pediatric cancer continuum: A systematic review. Int J Nurs Sci [Internet] Elsevier Ltd; 2021;8(1):22–29. PMID:33575441

119. Agarwal S, Glenton C, Henschke N, Tamrat T, Bergman H, Ms F, Gl M, Lewin S. mobile devices : a mixed methods systematic review ( Review ). 2020; [doi: 10.1002/14651858.CD012907.pub2.www.cochranelibrary.com]

120. Svendsen MJ, Wood KW, Kyle J, Cooper K, Rasmussen CDiN, Sandal LF, Stochkendahl MJ, Mair FS, Nicholl BI. Barriers and facilitators to patient uptake and utilisation of digital interventions for the self-management of low back pain: A systematic review of qualitative studies. BMJ Open 2020;10(12). PMID:33310794

121. Gilbey D, Morgan H, Lin A, Perry Y. Effectiveness, acceptability, and feasibility of digital health interventions for LGBTIQ+ young people: Systematic review. J Med Internet Res 2020;22(12). PMID:33270039

122. Robinson A, Oksuz U, Slight R, Slight S, Husband A. Digital and mobile technologies to promote physical health behavior change and provide psychological support for patients undergoing elective surgery: Meta-ethnography and systematic review. JMIR mHealth uHealth 2020;8(12):1–15. PMID:33258787

123. Turan Kavradim S, Yangöz ŞT, Canli Ozer Z, Boz I. Instruments to assess self-efficacy among people with cardiovascular disease: A COSMIN systematic review. Int J Clin Pract 2020;74(11):1–15. PMID:33166049

124. Seppen BF, Den Boer P, Wiegel J, ter Wee MM, Van der Leeden M, De Vries R, Van der Esch M, Bos WH. Asynchronous mhealth interventions in rheumatoid arthritis: Systematic scoping review. JMIR mHealth uHealth 2020;8(11):1–11. PMID:33151161

125. Farnood A, Johnston B, Mair FS. A mixed methods systematic review of the effects of patient online self-diagnosing in the “smart-phone society” on the healthcare professional-patient relationship and medical authority. BMC Med Inform Decis Mak BMC Medical Informatics and Decision Making; 2020;20(1):1–14. PMID:33023577

126. Godinho MA, Jonnagaddala J, Gudi N, Islam R, Narasimhan P, Liaw ST. mHealth for Integrated People-Centred Health Services in the Western Pacific: A Systematic Review. Int J Med Inform [Internet] Elsevier B.V.; 2020;142(July):104259. PMID:32858339

127. Allman-Farinelli M, Chen J, Chevance G, Partridge SR, Gemming L, Patrick K, Godino JG. The efficacy of electronic health interventions targeting improved sleep for achieving prevention of weight gain in adolescents and young to middle-aged adults: A systematic review. Obes Rev 2020;21(6):1–17. PMID:32037672

128. Tønning ML, Kessing LV, Bardram JE, Faurholt-Jepsen M. Methodological challenges in randomized controlled trials on smartphone-based treatment in psychiatry: Systematic review. J Med Internet Res 2019;21(10):1–22. PMID:31663859

129. Trifan A, Oliveira M, Oliveira JL. Passive sensing of health outcomes through smartphones: Systematic review of current solutions and possible limitations. JMIR mHealth uHealth 2019;7(8). PMID:31444874

130. Haddad SM, Souza RT, Cecatti JG. Mobile technology in health (mHealth) and antenatal care–Searching for apps and available solutions: A systematic review. Int J Med Inform [Internet] Elsevier; 2019;127(December 2018):1–8. PMID:31128820

131. Masè M, Micarelli A, Strapazzon G. Hearables: New Perspectives and Pitfalls of In-Ear Devices for Physiological Monitoring. A Scoping Review. Front Physiol 2020;11(October):1–18. [doi: 10.3389/fphys.2020.568886]

132. Small SR, Bullock GS, Khalid S, Barker K, Trivella M, Price AJ. Current clinical utilisation of wearable motion sensors for the assessment of outcome following knee arthroplasty: a scoping review. BMJ Open 2019;9(12). PMID:31888943

133. Teshuva I, Hillel I, Gazit E, Giladi N, Mirelman A, Hausdorff JM. Using wearables to assess bradykinesia and rigidity in patients with Parkinson’s disease: a focused, narrative review of the literature. J Neural Transm [Internet] Springer Vienna; 2019;126(6):699–710. PMID:31115669

134. Bentley KH, Kleiman EM, Elliott G, Huffman JC, Nock MK. Real-time monitoring technology in single-case experimental design research: Opportunities and challenges. Behav Res Ther 2019;117(November 2018):87–96. PMID:30579623

135. Chieng D, Paul V, Denman R. Current Device Therapies for Sudden Cardiac Death Prevention – the ICD, Subcutaneous ICD and Wearable ICD. Hear Lung Circ [Internet] Australian and New Zealand Society of Cardiac and Thoracic Surgeons (ANZSCTS) and the Cardiac Society of Australia and New Zealand (CSANZ); 2019;28(1):65–75. PMID:30389367

136. Henschke N, Bs B, Gl M, Tamrat T, Shepperd S, Dc G, Ar JM, Villanueva G, Ms F, Glenton C, Lewin S, Henschke N, Bs B. Gonçalves-Bradley DC, J Maria AR, Ricci-Cabello I, Villanueva G, Fønhus MS, Glenton C, Lewin S, Henschke N, Buckley BS, Mehl GL, Tamrat T, Shepperd S. 2020; [doi: 10.1002/14651858.CD012927.pub2.www.cochranelibrary.com]

137. Odendaal WA, Anstey Watkins J, Leon N, Goudge J, Griffiths F, Tomlinson M, Daniels K. Health workers’ perceptions and experiences of using mHealth technologies to deliver primary healthcare services: a qualitative evidence synthesis. Cochrane Database Syst Rev [Internet] 2020 Mar 26; [doi: 10.1002/14651858.CD011942.pub2]

138. McHenry MS, Fischer LJ, Chun Y, Vreeman RC. A systematic review of portable electronic technology for health education in resource-limited settings. Glob Health Promot 2019;26(2):70–81. PMID:28832243

139. MOSKOVITCH J, VOSKOBOINIK A. Cardiac resynchronization therapy: a comprehensive review. Minerva Med 2019;110(2):121–38.

140. Vienne-Jumeau A, Quijoux F, Vidal PP, Ricard D. Value of gait analysis for measuring disease severity using inertial sensors in patients with multiple sclerosis: Protocol for a systematic review and meta-analysis. Syst Rev Systematic Reviews; 2019;8(1):1–5. PMID:30621765

141. Thordardottir B, Malmgren Fänge A, Lethin C, Rodriguez Gatta D, Chiatti C. Acceptance and Use of Innovative Assistive Technologies among People with Cognitive Impairment and Their Caregivers: A Systematic Review. Biomed Res Int 2019;2019. PMID:30956989

142. De Farias FACD, Dagostini CM, Bicca YDA, Falavigna VF, Falavigna A. Remote patient monitoring: A systematic review. Telemed e-Health 2020;26(5):576–583. PMID:31314689

143. Liverpool S, Mota CP, Sales CMD, Čuš A, Carletto S, Hancheva C, Sousa S, Cerón SC, Moreno-Peral P, Pietrabissa G, Moltrecht B, Ulberg R, Ferreira N, Edbrooke-Childs J. Engaging children and young people in digital mental health interventions: Systematic review of modes of delivery, facilitators, and barriers. J Med Internet Res 2020;22(6). PMID:32442160

144. Sana F, Isselbacher EM, Singh JP, Heist EK, Pathik B, Armoundas AA. Wearable Devices for Ambulatory Cardiac Monitoring: JACC State-of-the-Art Review. J Am Coll Cardiol 2020;75(13):1582–1592. PMID:32241375

145. Shan R, Sarkar S, Martin SS. Digital health technology and mobile devices for the management of diabetes mellitus: state of the art. Diabetologia Diabetologia; 2019;62(6):877–887. PMID:30963188

146. Milne-Ives M, LamMEng C, de Cock C, van Velthoven MH, Ma EM. Mobile apps for health behavior change in physical activity, diet, drug and alcohol use, and mental health: Systematic review. JMIR mHealth uHealth 2020;8(3):1–16. PMID:32186518

147. McCann L, McMillan KA, Pugh G. Digital interventions to support adolescents and young adults with cancer: Systematic review. JMIR Cancer 2019;5(2). [doi: 10.2196/12071]

148. Brown A, O’Connor S. Mobile health applications for people with dementia: a systematic review and synthesis of qualitative studies. Informatics Heal Soc Care [Internet] Taylor & Francis; 2020;45(4):343–359. PMID:32237937

149. Van Rhoon L, Byrne M, Morrissey E, Murphy J, McSharry J. A systematic review of the behaviour change techniques and digital features in technology-driven type 2 diabetes prevention interventions. Digit Heal 2020;6:1–27. [doi: 10.1177/2055207620914427]

150. Böhm B, Karwiese SD, Böhm H, Oberhoffer R. Effects of mobile health including wearable activity trackers to increase physical activity outcomes among healthy children and adolescents: Systematic review. JMIR mHealth uHealth 2019;7(4). [doi: 10.2196/mhealth.8298]

151. Ballin M, Hult A, Björk S, Dinsmore J, Nordström P, Nordström A. Digital exercise interventions for improving measures of central obesity: a systematic review. Int J Public Health 2020;65(5):593–605. PMID:32410008

152. Hossain MM, Tasnim S, Sharma R, Sultana A, Shaik AF, Faizah F, Kaur R, Uppuluri M, Sribhashyam M, Bhattacharya S. Digital interventions for people living with non-communicable diseases in India: A systematic review of intervention studies and recommendations for future research and development. Digit Heal 2019;5:1–18. [doi: 10.1177/2055207619896153]

153. Azhar A, Gillani S, Mohiuddin G, Majeed R. A systematic review on clinical implication of continuous glucose monitoring in diabetes management. J Pharm Bioallied Sci [Internet] 2020;12(2):102. [doi: 10.4103/jpbs.JPBS_7_20]

154. Tsivgoulis G, Katsanos AH, Köhrmann M, Caso V, Perren F, Palaiodimou L, Deftereos S, Giannopoulos S, Ellul J, Krogias C, Mavridis D, Triantafyllou S, Alexandrov AW, Schellinger PD, Alexandrov A V. Duration of implantable cardiac monitoring and detection of atrial fibrillation in ischemic stroke patients: A systematic review and meta-analysis. J Stroke 2019;21(3):302–311. [doi: 10.5853/jos.2019.01067]

155. O’Driscoll R, Turicchi J, Beaulieu K, Scott S, Matu J, Deighton K, Finlayson G, Stubbs J. How well do activity monitors estimate energy expenditure? A systematic review and meta-analysis of the validity of current technologies. Br J Sports Med 2020;54(6):332–340. PMID:30194221

156. Chew D, Somayaji R, Conly J, Exner D, Rennert-May E. Timing of device reimplantation and reinfection rates following cardiac implantable electronic device infection: A systematic review and meta-analysis. BMJ Open 2019;9(9):1–9. PMID:31481556

157. Zhang Y, Weaver RG, Armstrong B, Burkart S, Zhang S, Beets MW. Validity of Wrist-Worn photoplethysmography devices to measure heart rate: A systematic review and meta-analysis. J Sports Sci [Internet] Routledge; 2020;38(17):2021–2034. PMID:32552580

158. Kanitsoraphan C, Rattanawong P, Mekraksakit P, Chongsathidkiet P, Riangwiwat T, Kanjanahattakij N, Vutthikraivit W, Klomjit S, Thavaraputta S. Baseline fragmented QRS is associated with increased all-cause mortality in heart failure with reduced ejection fraction: A systematic review and meta-analysis. Ann Noninvasive Electrocardiol [Internet] 2019 Mar;24(2):e12597. [doi: 10.1111/anec.12597]

159. Kamei T, Kanamori T, Yamamoto Y, Edirippulige S. The use of wearable devices in chronic disease management to enhance adherence and improve telehealth outcomes: A systematic review and meta-analysis. J Telemed Telecare 2020; [doi: 10.1177/1357633X20937573]

160. Petraglia F, Scarcella L, Pedrazzi G, Brancato L, Puers R, Costantino C. Inertial sensors versus standard systems in gait analysis: A systematic review and meta-analysis. Eur J Phys Rehabil Med 2019;55(2):265–280. PMID:30311493

161. Gandolla M, Antonietti A, Longatelli V, Pedrocchi A. The Effectiveness of Wearable Upper Limb Assistive Devices in Degenerative Neuromuscular Diseases: A Systematic Review and Meta-Analysis. Front Bioeng Biotechnol 2020;7(January):1–16. [doi: 10.3389/fbioe.2019.00450]

162. Zhou C, Hu H, Wang C, Zhu Z, Feng G, Xue J, Yang Z. The effectiveness of mHealth interventions on postpartum depression: A systematic review and meta-analysis. J Telemed Telecare 2022;28(2):83–95. PMID:32306847

163. Yang F, Wang J, Li W, Xu Y, Wan K, Zeng R, Chen Y. The prognostic value of late gadolinium enhancement in myocarditis and clinically suspected myocarditis: systematic review and meta-analysis. Eur Radiol European Radiology; 2020;30(5):2616–2626. PMID:32040731

164. Mai HN, Kim J, Choi YH, Lee DH. Accuracy of portable face-scanning devices for obtaining three-dimensional face models: A systematic review and meta-analysis. Int J Environ Res Public Health 2021;18(1):1–15. PMID:33375533

165. Lima da Silva G, Nunes-Ferreira A, Cortez-Dias N, de Sousa J, J. Pinto F, Caldeira D. Radiofrequency catheter ablation of ventricular tachycardia in ischemic heart disease in light of current practice: a systematic review and meta-analysis of randomized controlled trials. J Interv Card Electrophysiol Journal of Interventional Cardiac Electrophysiology; 2020;59(3):603–616. PMID:32948937

166. Wu X, Zhang T, Chen L, Chen X. Prolonging the flush-lock interval of totally implantable venous access ports in patients with cancer: A systematic review and meta-analysis. J Vasc Access 2021;22(5):814–821. PMID:32873129

167. Mao YJ, Wang H, Chen JX, Huang PF. Meta-analysis of medical management versus catheter ablation for atrial fibrillation. Rev Cardiovasc Med 2020;21(3):419–432. PMID:33070546

168. Turagam MK, Garg J, Whang W, Sartori S, Koruth JS, Miller MA, Langan N, Sofi A, Gomes A, Choudry S, Dukkipati SR. Annals of Internal Medicine R EVIEW Catheter Ablation of Atrial Fibrillation in Patients With Heart Failure. 2018; [doi: 10.7326/M18-0992]

169. Basu-ray I, Bunch TJ, Gold M, Saeed M. Meta-analysis comparing outcomes of catheter ablation for ventricular arrhythmia in ischemic versus nonischemic cardiomyopathy. 2021;(October 2020):54–62. [doi: 10.1111/pace.14129]

170. Schmidt-lucke C, Käferle J, Berner BR, Ahlborg L, Hansen HM, Tollefsen US, Thon T, Moen RD, Pekanovic A, Tornberg ÅB, Lauruschkus K. Effect of assisted walking-movement in patients with genetic and acquired neuromuscular disorders with the motorised Innowalk device : an international case study meta-analysis. 2019; [doi: 10.7717/peerj.7098]

171. Bajraktari G, Bytyçi I, Henein MY. Left atrial structure and function predictors of recurrent fibrillation after catheter ablation: a systematic review and meta‐analysis. Clin Physiol Funct Imaging [Internet] 2020 Jan 21;40(1):1–13. [doi: 10.1111/cpf.12595]

172. Kedrzycki MS, Leiloglou M, Ashrafian H, Jiwa N, Thiruchelvam PTR, Elson DS, Leff DR, Hons MS. Meta-analysis Comparing Fluorescence Imaging with Radioisotope and Blue Dye-Guided Sentinel Node Identification for Breast Cancer Surgery. Ann Surg Oncol [Internet] Springer International Publishing; 2021;3738–3748. [doi: 10.1245/s10434-020-09288-7]

173. Ramdas WD. Efficacy of glaucoma drainage devices in uveitic glaucoma and a meta-analysis of the literature. Graefe’s Archive for Clinical and Experimental Ophthalmology; 2019;143–151.

174. Li H, Cao L, Li X, Shi X, Liu X, Yang K. Complementary Therapies in Medicine The e ff ectiveness and safety of acupuncture for depression : An overview of. 2020;50(June 2019). [doi: 10.1016/j.ctim.2019.102202]

175. Zhang N, Hou L, Yan P, Li X, Wang Y, Niu J, Feng L, Li J, Yang K, Liu X. Electro-acupuncture vs. sham electro-acupuncture for chronic severe functional constipation: A systematic review and meta-analysis. Complement Ther Med [Internet] Elsevier; 2020 Nov;54(January):102521. [doi: 10.1016/j.ctim.2020.102521]

176. Hodkinson A, Kontopantelis E, Adeniji C, van Marwijk H, McMillan B, Bower P, Panagioti M. Notice of Retraction. Hodkinson et al. Accelerometer- and Pedometer-Based Physical Activity Interventions Among Adults With Cardiometabolic Conditions: A Systematic Review and Meta-analysis. JAMA Netw Open . 2019;2(10):e1912895. JAMA Netw Open [Internet] 2020 Dec 18;3(12):e2032700. [doi: 10.1001/jamanetworkopen.2020.32700]
