# Supplementary material for: Digital Biomarker–Based Interventions: Systematic Review of Systematic Reviews
Source: J Med Internet Res. 2022 Dec 21;24(12):e41042. doi: 10.2196/41042 (PMC9813819; doi:10.2196/41042)
Supplement: Multimedia Appendix 4 [file jmir_v24i12e41042_app4.docx]

Characteristics of the included studies.

| Digital biomarker (behavioral, physiological data/ digital device) | Role of digital biomarker | Author, year, and country | No. of included studies in qualitative and quantitative synthesis | Study designs | Participants: diagnosis (age range) | Intervention | Comparator | Outcome and number of included studies |
| --- | --- | --- | --- | --- | --- | --- | --- | --- |
| Heart rhythm/ CRT(D)/ICD ^e^ | Influencing intervention | Liu, Y. et al. (2020) [63]/China | 26 | RCT^a^, C^c^, CC^d^ | Chronic kidney disease, Kidney failure, (adults) | Conversion of cardiac rhythm | Control group | All-cause mortality (7) |
|  |  |  |  |  |  |  |  | non-primary prevention (5) |
| Gait pattern functions, Looking after one's health, Weight maintenance functions/ Fitbit | Influencing intervention | Ringeval, M. et al. (2020) [57]/ Canada | 37 | RCT | Diseases of the circulatory system, Chronic obstructive pulmonary disease, Employees, Students, Healthy subjects (adult, elderly) | Physical activity behaviours, Weight maintenance functions | Control group | steps per day (16) |
|  |  |  |  |  |  |  |  | Moderate-to-vigorous physical activity (14) |
|  |  |  |  |  |  |  |  | weight (11) |
|  |  |  |  |  |  |  |  | Sedentary behaviors (sitting time, min/day)-objectively measured (4) |
|  |  |  |  |  |  |  |  | Sedentary behaviors (sitting time, min/day)- self reported (2) |
| Heart functions / ICD (implantable cardioverter-defibrillators), iPhone-based rhythm monitoring device, pacemakers | Influencing intervention | Jang, J. et al. (2020) [55]  / Taiwan | 32 | RCT | Pacemaker or implantable cardioverter defibrillator complication, Presence of cardiac resynchronization therapy defibrillator (adult/elderly) | Cardiac electrophysiological monitoring | in-office (IO) follow-up | Detection rate of atrial arrhythmia (14) |
|  |  |  |  |  |  |  |  | The incidence of stroke (7) |
| Heart functions/ Metronome with a siren, HeartStart-MRx, Zoll AED, Cardio First AngelTM, | Influencing intervention | Wang, S. et al. (2020) [56]  / Taiwan | 11 | RCT, non-RCT^b^ | Cardiac arrest (adult) | Cardiopulmonary resuscitation | usual care | Return of spontaneous circulation (ROSC) (11) |
|  |  |  |  |  |  |  |  | Survival-to-discharge (7) |
| Looking after one's health, Running, Walking/ activity monitor, portable tablet computers with touch screens, Fitbit, Jawbone UP24 wearable device, pedometer, accelerometer | Influencing intervention | Liu, YJ. et al. (2020) [59]  /Hong Kong | 10 | RCT | Lack of physical exercise (adult) | Physical activity behaviours | Without a device | Steps per day (2) |
|  |  |  |  |  |  |  |  | Moderate-to-vigorous physical activity (MVPA) (3) |
| Walking, Weight maintenance functions/ Fitbit, Jawbone Up24, Combined HR monitor and accelerometer (Actiheart), Wrist-worn accelerometer, FIT Core, Body Media, Fitbug Orb, Polar FA20 accelerometer | Influencing intervention | Tang, MSS, et al. (2020) [49]/ Australia | 12 | RCT | General population, (adult) | Physical activity behaviours, Weight maintenance functions | Control group | Physical activity (total steps, total activity,  the proportion of participants at activity goal) (12) |
|  |  |  |  |  |  |  |  | Steps per day (7) |
|  |  |  |  |  |  |  |  | weight (4) |
|  |  |  |  |  |  |  |  | physical activity (total steps, total activity,  the proportion of participants at activity goal) in overweight adults (5) |
|  |  |  |  |  |  |  |  | Physical activity (total steps, total activity,  the proportion of participants at activity goal) on healthy adults (7) |
| Walking/ wearable activity trackers | Influencing intervention | Franssen, WMA. et al. (2020) [62]  /Belgium | 35 | RCT | Chronic respiratory failure, Type 2 diabetes mellitus, Diseases of the circulatory system, Overweight in adults, (adult) | Physical activity behaviours | usual activity | Steps per day in Chronic respiratory diseases (8) |
|  |  |  |  |  |  |  |  | Steps per day in Type 2 diabetes mellitus (12) |
|  |  |  |  |  |  |  |  | Steps per day in Cardiovascular diseases (6) |
|  |  |  |  |  |  |  |  | Steps per day in Overweight/Obesity (2) |
|  |  |  |  |  |  |  |  | Steps per day in Sedentary older adults (8) |
|  |  |  |  |  |  |  |  | Steps per day (36) |
| Looking after one's health, Walking/Accelerometer, pedometer | Influencing intervention | Kwan, RYC. et al. (2020) [60]/Hong Kong | 38 | RCT | Obstructive sleep apnoea, Diabetes mellitus, Diseases of the circulatory system, , Chronic obstructive pulmonary disease, Overweight, obesity or specific nutrient excesses, Parkinson disease, Healthy subjects (adult) | Physical activity behaviours | usual care | physical activity (time spent on physical activity,  energy expended on physical activity, step counts,  sedentary time) time measured by questionnaires (9) |
|  |  |  |  |  |  |  |  | physical activity (time spent on physical activity,  energy expended on physical activity, step counts,  sedentary time) time measured by objective wearable devices (5) |
|  |  |  |  |  |  |  |  | Energy expenditure (4) |
|  |  |  |  |  |  |  |  | Steps per day (9) |
| Heart rhythm/implantable cardioverter defibrillator (ICD) | Influencing intervention | Gama, F. et al. (2020) [68]  /Portugal | 11 | RCT | Left ventricular failure with reduced ejection fraction (adult) | Conversion of cardiac rhythm | without ICD | All-cause death (8) |
|  |  |  |  |  |  |  |  | Sudden cardiac death (6) |
| Heart rhythm/ implantable cardioverter defibrillator (ICD) | Influencing intervention | Alotaibi, S. et al (2020) [69]  /Saudi Arabia | 13 | RCT | Heart failure (adult, elderly) | Conversion of cardiac rhythm | standard of care | All-cause mortality (7) |
|  |  |  |  |  |  |  |  | heart failure related hospitalization (12) |
| Looking after one's health, Walking/Fitbit, Jawbone UP, Polar Active, Misfit Flash, Gruve Solution, LUMOback, BodyMedia Fit, SenseWear, ActiveLink, InBodyBand | Influencing intervention | Lynch, C. et al. (2019) [47]  /Australia | 21 | RCT | Neoplasms, Overweight, obesity or specific nutrient excesses, Lack of physical exercise, Malignant neoplasms of breast, Osteoarthritis, Chronic arterial occlusive disease, Other specified disorders with neurocognitive impairment as a major feature (adult) | Physical activity behaviours | Control group, alternative intervention | Moderate-to-vigorous physical activity versus control (5) |
|  |  |  |  |  |  |  |  | Steps per day versus control (7) |
|  |  |  |  |  |  |  |  | moderate-to-vigorous physical activity versus alternative intervention (7) |
|  |  |  |  |  |  |  |  | Steps per day versus an alternative intervention (6) |
| Heart rhythm/ Cardio MEMS, RVP sensor, Chronicle, ICD-OptiVol, InSync Sentry, lung impedance | Influencing intervention | Halawa, A. et al. (2019) [51]  / United States of America | 14 | RCT, C, CC | Heart failure (adult, elderly) | Conversion of cardiac rhythm | standard heart failure therapy | Pressure sensors for heart failure related readmission rate (4) |
|  |  |  |  |  |  |  |  | Impedance devices sensors for heart failure related readmission rate (9) |
|  |  |  |  |  |  |  |  | Pressure sensors and Impedance devices sensors for heart failure related readmission rate (13) |
|  |  |  |  |  |  |  |  | Pressure sensors for all-cause mortality (4) |
|  |  |  |  |  |  |  |  | Impedance devices sensors for all-cause mortality (9) |
|  |  |  |  |  |  |  |  | Pressure sensors and Impedance devices sensors for all-cause mortality (13) |
|  |  |  |  |  |  |  |  | Pressure sensors for Combined HF related readmission and all-cause death (4) |
|  |  |  |  |  |  |  |  | Impedance devices for Combined HF related readmission and all-cause death (8) |
|  |  |  |  |  |  |  |  | Pressure sensors and Impedance devices for Combined HF related readmission and all-cause death (12) |
| Walking, Aerobic capacity/ Garmin, Pedometer, Fitbit, Accelerometer, Yamax Digiwalker, Gex sensor of vital signs and smartphone, | Influencing intervention | Hannan, AL. et al. (2019) [48]  / Australia | 9 | RCT | Diseases of the circulatory system (adult, elderly) | Assisting and leading exercise for exercise tolerance function, Physical activity behaviours | control group | Aerobic capacity (3) |
|  |  |  |  |  |  |  |  | Steps per day (4) |
| Weight maintenance functions/ wristbands, smartwatches | Influencing intervention | Yen, H. et al. (2019) [54]  /Taiwan | 19 | RCT | General population (adult) | Weight maintenance functions, Whole body measurement, Body measurement of trunk | without wearables | weight (19) |
|  |  |  |  |  |  |  |  | Body-mass index (12) |
|  |  |  |  |  |  |  |  | Waist circumference (4) |
| Walking, Looking after one's health/ Fitbit, Jawbone UP24, Gruve, LumoBack, Polar Active, Fitbug, Pebble+, Fitmeter, Personal Activity Monitor, Withings Pulse | Influencing intervention | Brickwood, K. et al. (2019) [46]  / Australia | 28 | RCT | General population (adult) | Physical activity behaviours | without a device | Steps per day (5) |
|  |  |  |  |  |  |  |  | Moderate to vigorous physical activity (3) |
|  |  |  |  |  |  |  |  | Energy expenditure (9) |
|  |  |  |  |  |  |  |  | Sedentary behaviors (sitting time) (3) |
| Looking after one's health, Walking/ Accelerometer, Dynaport MoveMonitor, Pedometer, Yamax Digi-walker CW700, ActivPal, ActiGraph, Personal Activity Monitor | Influencing intervention | Braakhuis, HEM. et al. (2019) [67]  / the Netherlands | 14 | RCT | Stroke not known if ischaemic or haemorrhagic, Diseases of coronary artery, Cardiac rehabilitation, Chronic obstructive pulmonary disease, Lack of physical exercise, Parkinsonism, Heart failure, Presence of cardioverter-defibrillator, Diabetes mellitus (adult) | Physical activity behaviours | Control group | physical activity (steps per day, energy expenditure, walking time) (12) |
| Looking after one's health/ Fitbit, Yorbody, AiperMotion | Influencing intervention | Kirk, MA. et al. (2019) [58]  /Canada | 35 | RCT | Metabolic disorders (adult) | Physical activity behaviours | Exercise | Steps per day (19) |
|  |  |  |  |  |  |  |  | Moderate to vigorous physical activity (15) |
| Looking after one's health, Walking/ accelerometer, pedometers, Yamax, Fitbit, | Influencing intervention | Davergne T. et al. (2019) [64]  /France | 17 | RCT, C | Rheumatism, Diseases of the musculoskeletal system or connective tissue (adult) | Physical activity behaviours, Pain, Test of functions, Quality of life, Test of muscle endurance | usual care | Steps per day (7) |
|  |  |  |  |  |  |  |  | Moderate to vigorous physical activity (3) |
|  |  |  |  |  |  |  |  | Pain (7) |
|  |  |  |  |  |  |  |  | Disability (5) |
|  |  |  |  |  |  |  |  | Functional tests (4) |
|  |  |  |  |  |  |  |  | Quality of life (4) |
|  |  |  |  |  |  |  |  | Fatigue (1) |
| Heart rhythm/ Fragmented QRS (fQRS) | Influencing intervention | Kanitsoraphan, C. et al. (2019) [70]/ Thailand | 10 | C | Left ventricular failure (elderly) | Cardiac electrophysiological monitoring | Control group | Major arrhythmic events in ICD patients (5) |
|  |  |  |  |  |  |  |  | Major arrhythmic events in no ICD patients (5) |
|  |  |  |  |  |  |  |  | All-cause mortality in patients with ejection fraction < 35% (9) |
|  |  |  |  |  |  |  |  | All-cause mortality in patients with nonspecific ejection fraction (2) |
|  |  |  |  |  |  |  |  | All-cause mortality in patients with ejection fraction < 35% and nonspecific ejection fraction (11) |
|  |  |  |  |  |  |  |  | Major arrhythmic events in patients with ejection fraction < 35% (9) |
|  |  |  |  |  |  |  |  | Major arrhythmic events in patients with nonspecific ejection fraction (2) |
|  |  |  |  |  |  |  |  | Major arrhythmic events in patients with ejection fraction < 35 % and nonspecific ejection fraction (11) |
| Heart rhythm/ implantable cardiac defibrillator (ICD) | Influencing intervention | Elkaryoni, A. et al. (2019) [53]/ the united states of America | 8 | C | Cardiovascular devices associated with injury or harm, left ventricular assist devices, Heart failure (elderly) | Conversion of cardiac rhythm | Control group | All-cause mortality for ICD use in patients with a CF-LVAD (8) |
|  |  |  |  |  |  |  |  | All-cause mortality for ICDs implanted post-CF-LVAD (2) |
|  |  |  |  |  |  |  |  | likelihood of transplant associated with ICD use in patients with a CF-LVAD (5) |
| Involuntary movement reaction functions/ accelerometer-based navigation system | Influencing intervention | Shigemura, T. et al. (2019) [66]/ Japan | 10 | RCT | Injuries to the knee or lower leg (elderly) | Body measurement of lower limb, Test of maintaining body position | Control group | Lower limb mechanical axis outliers (14) |
|  |  |  |  |  |  |  |  | coronal femoral component alignment outliers (18) |
|  |  |  |  |  |  |  |  | Coronal tibial component alignment (16) |
| Heart functions/ implantable cardiac monitor, Holter-Electrocardiogram | Influencing intervention | Tsivgoulis, G. et al. (2019) [65]  /Greece | 4 | RCT, C | Cerebral ischaemic stroke (elderly) | Cardiac electrophysiological monitoring | conventional (non-PCM) cardiac monitoring | Detection of paroxysmal  atrial fibrillation (4) |
|  |  |  |  |  |  |  |  | probability of anticoagulant  initiation during follow-up (3) |
|  |  |  |  |  |  |  |  | Risk of recurrent  stroke (4) |
| Walking and moving/ pedometer | Influencing intervention | Armstrong, M. et al. (2019) [61]  / the United Kingdom of Great Britain and Northern Ireland | 17 | RCT | Chronic obstructive pulmonary disease (elderly) | Physical activity behaviours | without physical activity promotion intervention | Steps per day, pedometer-based physical activity (PA) promotion (12) |
|  |  |  |  |  |  |  |  | Steps per day, Pedometer PA promotion + pulmonary rehabilitation promotion (7) |
| Heart rhythm/ Cardiac resynchronization therapy, ICD | Influencing intervention | Voruganti, DC. et al. (2019) [52]/the United States of America | 7 | RCT, C | Cardiovascular devices associated with injury or harm, left ventricular assist devices. (Adult/ elderly) | Conversion of cardiac rhythm | Control group | Incidence of ventricular arrhythmia (4) |
|  |  |  |  |  |  |  |  | All-cause mortality (3) |
|  |  |  |  |  |  |  |  | Incidence of ICD therapies (4) |
|  |  |  |  |  |  |  |  | Hospitalizations (2) |
| Looking after  one's health/ pedometer | Influencing intervention | Freak-Poli, RL. et al. (2020) [50]  /Australia | 14 | RCT | General population (adult) | Physical activity behaviours, Whole body measurement | without pedometer, physical activity | Physical activity (steps per day, metabolic equivalents, incidental activity): combined At completion of intervention (short duration, 1 week) (1) |
|  |  |  |  |  |  |  |  | Physical activity (steps per day, metabolic equivalents, incidental activity): combined At completion of intervention (medium duration, 3 to 6 months) (3) |
|  |  |  |  |  |  |  |  | Physical activity (steps per day, metabolic equivalents, incidental activity): combined Follow-up after completion (medium, 3 months) (1) |
|  |  |  |  |  |  |  |  | Body-mass index At completion of intervention (medium duration, 3 to 6 months) (2) |

^a^RCT: randomized controlled trial, ^b^non-RCT: non randomized controlled trial, ^c^C: cohort study, ^d^CC: case-control study, ^e^ Cardiac resynchronization therapy with or without a defibrillator (CRT(D)) and implantable cardioverter defibrillator
