# Supplementary material for: Digital Biomarker–Based Interventions: Systematic Review of Systematic Reviews
Source: J Med Internet Res. 2022 Dec 21;24(12):e41042. doi: 10.2196/41042 (PMC9813819; doi:10.2196/41042)
Supplement: Multimedia Appendix 6 [file jmir_v24i12e41042_app6.docx]

Evidence summary and quality assessment by the GRADE (Grading of Recommendations, Assessment, Development, and Evaluations) tool.

| Intervention/ ICF category | Outcome | | Author (year) | Number of patients total/ intervention | | Effect size (95% CI)/ measure | | Risk of bias | | | Imprecision | | | Inconsistency | | | Indirectness | | | Publication bias | | Grade overall quality rating | | Comments | | |  |  |
| --- | --- | --- | --- | --- | --- | --- | --- | --- | --- | --- | --- | --- | --- | --- | --- | --- | --- | --- | --- | --- | --- | --- | --- | --- | --- | --- | --- | --- |
| Implantable cardiac defibrillator (ICD) | | | | | | | | | | | | | | | | | | | | | | | | | | | |  |
| ICD^a^/ Heart rhythm | All-cause mortality | | Liu, Y. et al (2020) [63] | 12923/2515 | | 0.66 (1,1.26) OR^b^ | | | -1 | | | 0 | | | -1 | | | 0 | | 0 | | M ^l^ | | | None of the included studies have low risk of bias. High heterogeneity (92%). | |  |  |
| ICD/ Heart rhythm | All-cause mortality | | Alotaibi, S, et al (2020) [69] | 4460/not reported | | 0.88 (0.69,1.11) RR^c^ | | | -1 | | | 0 | | | 0 | | | 0 | | 0 | | M | | | 43% of the included studies have low risk of bias. | |  |  |
| ICD / Heart rhythm | All-cause mortality for ICDs implanted post-CF-LVAD^d^ | | Elkaryoni, A, et al (2019) [53] | 677/516 | | 0.87  (0.48,1.57) HR^e^ | | | 0 | | | 0 | | | 0 | | | 0 | | 0 | | H ^m^ | | |  | |  |  |
| ICD / Heart rhythm | All-cause mortality for ICD use in patients with a CF-LVAD | | Elkaryoni, A, et al (2019) [53] | 6388/3450 | | 0.96(0.73,1.27) HR | | | 0 | | | 0 | | | 0 | | | 0 | | 0 | | H | | |  | |  |  |
| ICD/ Heart rhythm | All-cause mortality | | Gama, F, et al (2020) [68] | 31701/3631 | | 0.85(0.78,0.94) RR | | | -1 | | | 0 | | | 0 | | | 0 | | 0 | | M | | | None of the included studies have low risk of bias. | |  |  |
| ICD/ Heart rhythm | sudden cardiac death | | Gama, F, et al (2020) [68] | 21977/2386 | | 0.49(0.4,0.61) RR | | | -1 | | | 0 | | | 0 | | | 0 | | 0 | | M | | | None of the included studies have low risk of bias. | |  |  |
| ICD/ Heart rhythm | Non-primary prevention | | Liu, Y. et al (2020) [63] | 6858/862 | | 0.47(0.4,0.55) OR | | | -1 | | | 0 | | | 0 | | | 0 | | 0 | | M | | | None of the included studies have low risk of bias. | |  |  |
| ICD/ Heart rhythm | Heart failure related hospitalization | | Alotaibi, S, et al (2020) [69] | 5565/not reported | | 0.95(0.78,0.16) RR | | | -1 | | | 0 | | | -1 | | | 0 | | 0 | | M | | | 58% of the included studies have low risk of bias. High heterogeneity (83.5%). | |  |  |
| ICD / Heart rhythm | likelihood of transplant associated with ICD use in patients with a CF-LVAD | | Elkaryoni, A, et al (2019) [53] | 5716/2917 | | 1.1(0.93,1.3) HR | | | 0 | | | 0 | | | 0 | | | 0 | | 0 | | H | | |  | |  |  |
| ICD, CRT, heart monitoring devices | | | | | | | | | | | | | | | | | | | | | | | | | | | | |
| ICD, iPhone-based rhythm monitoring device, pacemakers/ Heart functions | | detection rate of atrial arrhythmia | Jang, J. et al (2020) [55] | | 14547/6659 | | 1.363(1.147,1.619) RR | | | 0 | | | 0 | | | 0 | | | 0 | | 0 | | H | | |  |  |  |
| ICD, iPhone-based rhythm monitoring device, pacemakers/ Heart functions | | the incidence of stroke | Jang, J. et al (2020) [55] | | 4063/2254 | | 0.539(0.31,0.936) RR | | | 0 | | | 0 | | | 0 | | | 0 | | 0 | | H | | |  |  |  |
| Implantable cardiac monitor, Holter-Electrocardiogram/ Heart functions | | detection of paroxysmal atrial fibrillation | Tsivgoulis, G, et al (2019) [65] | | 1102/533 | | 2.46(1.61,3.76) RR | | | -1 | | | -1 | | | 0 | | | 0 | | 0 | | M | | | None of the included studies have low risk of bias. Optimal information size is 1140. |  |  |
| Implantable cardiac monitor, Holter-Electrocardiogram/ Heart functions | | probability of anticoagulant initiation during follow-up | Tsivgoulis, G, et al (2019) [65] | | 956/468 | | 2.07(1.36,3.17) RR | | | -1 | | | -1 | | | 0 | | | 0 | | 0 | | M | | | None of the included studies have low risk of bias. Optimal information size is 1368. |  |  |
| Implantable cardiac monitor, Holter-Electrocardiogram/ Heart functions | | risk of recurrent stroke | Tsivgoulis, G, et al (2019) [65] | | 1102/533 | | 0.45(0.21,0.97) RR | | | -1 | | | -1 | | | 0 | | | 0 | | 0 | | M | | | None of the included studies have low risk of bias. Optimal information size is 1394. |  |  |
| CRT^f^, ICD/ Heart rhythm | | incidence of ventricular arrhythmia | Voruganti, DC, et al (2019) [52] | | 864/463 | | 1.36(0.99,1.86) OR | | | -1 | | | 0 | | | 0 | | | 0 | | 0 | | M | | | 25% of the included studies have a low risk of bias. |  |  |
| CRT, ICD/ Heart rhythm | | all-cause mortality | Voruganti, DC, et al (2019) [52] | | 933/462 | | 1.21(0.9,1.63) OR | | | -1 | | | 0 | | | 0 | | | 0 | | 0 | | M | | | 67% of the included studies have a low risk of bias. |  |  |
| CRT, ICD/ Heart rhythm | | incidence of ICD therapies | Voruganti, DC, et al (2019) [52] | | 870/461 | | 1.08(0.51,2.3) OR | | | -1 | | | 0 | | | 0 | | | 0 | | 0 | | M | | | 25% of the included studies have a low risk of bias. |  |  |
| CRT, ICD/ Heart rhythm | | Hospitalizations | Voruganti, DC, et al (2019) [52] | | 186/87 | | 1.36(0.59,3.14) OR | | | -1 | | | -1 | | | 0 | | | 0 | | 0 | | M | | | None of the included studies have low risk of bias. Total sample size is 186. |  |  |
| Activity trackers | | | | | | | | | | | | | | | | | | | | | | | | | | | | |
| Fitbit/ Looking after one's health, walking | | steps | Ringeval, M. et al (2020) [57] | | 1200/614 | | 590.54(475.89,1425.18) MD ^g^ | | | -1 | | | 0 | | | 0 | | | 0 | | 0 | | M | | | None of the included studies have low risk of bias |  |  |
| Activity monitor, portable tablet computers with touch screens, Fitbit, Jawbone UP24 wearable device, pedometer, accelerometer/ Looking after one's health, Walking | | Steps | Liu, YJ. et al (2020) [59] | | 83/41 | | 1.23(0.75,1.7) SMD ^h^ | | | -1 | | | -1 | | | 0 | | | 0 | | 0 | | M | | | None of the included studies have low risk of bias. Total sample is 83. |  |  |
| Fitbit, Jawbone Up24, Combined HR monitor and accelerometer (Actiheart), Wrist-worn accelerometer, FIT Core, Body Media, Fitbug Orb, Polar FA20 accelerometer/ Looking after one's health, Walking | | Steps | Tang, MSS, et al (2020) [49] | | 602/not reported | | 0.332(0.161,0.504) SMD | | | -1 | | | 0 | | | 0 | | | 0 | | 0 | | M | | | None of the included studies have low risk of bias. |  |  |
| Accelerometer, pedometer/ Looking after one's health, Walking | | Steps | Kwan, RYC, et al (2020) [60] | | 866/444 | | 0.79(0.3,1.28) MD | | | -1 | | | 0 | | | 0 | | | 0 | | 0 | | M | | | None of the included studies have low risk of bias. |  |  |
| Garmin, Pedometer, Fitbit, Accelerometer, Yamax Digiwalker, Gex sensor of vital signs and smartphone/ Looking after one's health, Walking | | Steps | Hannan, A, et al (2019) [48] | | 341/176 | | 0.45(-0.17,1.07) SMD | | | -1 | | | 0 | | | -1 | | | 0 | | 0 | | M | | | None of the included studies have low risk of bias.  High heterogeneity (81%). |  |  |
| Fitbit, Jawbone UP24, Gruve, LumoBack, Polar Active, Fitbug, Pebble+, Fitmeter, Personal Activity Monitor, Withings Pulse/ Looking after one's health, Walking | | Steps | Brickwood, K, et al (2019) [46] | | 934/470 | | 0.2(0.08,0.33) SMD | | | -1 | | | 0 | | | 0 | | | 0 | | 0 | | M | | | None of the included studies have low risk of bias. |  |  |
| Fitbit, Jawbone UP, Polar Active, Misfit Flash, Gruve Solution, LUMOback, BodyMedia Fit, SenseWear, ActiveLink, InBodyBand/ Looking after one's health, Walking | | Steps versus control | Lynch, C, et al (2019) [47] | | 800/not reported | | 0.25(0.17,0.32) SMD | | | -1 | | | 0 | | | 0 | | | 0 | | 0 | | M | | | None of the included studies have low risk of bias. |  |  |
| Fitbit, Jawbone UP, Polar Active, Misfit Flash, Gruve Solution, LUMOback, BodyMedia Fit, SenseWear, ActiveLink, InBodyBand/ Looking after one's health, Walking | | Steps versus an alternative intervention | Lynch, C, et al (2019) [47] | | 313/not reported | | -0.05(-0.22,0.13) SMD | | | -1 | | | -1 | | | 0 | | | 0 | | 0 | | M | | | None of the included studies have low risk of bias. Optimal information size is 788. |  |  |
| Fitbit, Yorbody, AiperMotion/ Looking after one's health, Walking | | Steps | Kirk, MA, et al (2019) [58] | | 1471/750 | | 2592.33(1688.62,3496.04) MD | | | -1 | | | 0 | | | -1 | | | 0 | | 0 | | M | | | None of the included studies have low risk of bias. High heterogeneity (91%). |  |  |
| accelerometer, pedometers, Yamax, Fitbit/ Looking after one's health, Walking | | Steps | Davergne T, et al (2019) [64] | | 463/238 | | 0.83(0.29,1.38) SMD | | | -1 | | | 0 | | | -1 | | | 0 | | 0 | | M | | | None of the included studies have low risk of bias. High heterogeneity (85%). |  |  |
| wearable activity trackers (pedometer)/ Looking after one's health, Walking | | Steps in Chronic respiratory diseases | Franssen, WMA, et al (2020) [62] | | 635/329 | | 1314(203,2426) MD | | | -1 | | | -1 | | | -1 | | | 0 | | 0 | | L ^n^ | | | None of the included studies have low risk of bias. Optimal information size is 788. High heterogeneity (92.3%). |  |  |
| wearable activity trackers (pedometer)/ Looking after one's health, Walking | | Steps in Type 2 diabetes mellitus | Franssen, WMA, et al (2020) [62] | | 706/390 | | 2693(1804,3581) MD | | | -1 | | | 0 | | | -1 | | | 0 | | 0 | | M | | | None of the included studies have low risk of bias.  High heterogeneity (86.2%). |  |  |
| wearable activity trackers (pedometer)/ Looking after one's health, Walking | | Steps in Cardiovascular diseases | Franssen, WMA, et al (2020) [62] | | 576/277 | | 1300(370,2230) MD | | | -1 | | | -1 | | | 0 | | | 0 | | 0 | | M | | | None of the included studies have low risk of bias. Optimal information size is 788. |  |  |
| wearable activity trackers (pedometer)/ Looking after one's health, Walking | | Steps in Overweight,Obesity | Franssen, WMA, et al (2020) [62] | | 154/79 | | 2405(1232,3577) MD | | | -1 | | | -1 | | | -1 | | | 0 | | 0 | | L | | | None of the included studies have low risk of bias. Total sample size is 154. High heterogeneity (77.1%) |  |  |
| wearable activity trackers (pedometer)/ Looking after one's health, Walking | | Steps in Sedentary older adults | Franssen, WMA, et al (2020) [62] | | 710/360 | | 2568(1396,3740) MD | | | -1 | | | -1 | | | -1 | | | 0 | | 0 | | L | | | None of the included studies have low risk of bias. Optimal information size is 788. High heterogeneity (82.1%). |  |  |
| wearable activity trackers (pedometer)/ Looking after one's health, Walking | | Steps (in total) | Franssen, WMA, et al (2020) [62] | | 2774/1435 | | 2123(1605,2641) MD | | | -1 | | | 0 | | | -1 | | | 0 | | 0 | | M | | | None of the included studies have low risk of bias. High heterogeneity (92.3%). |  |  |
| Pedometer PA promotion + pulmonary rehabilitation promotion/ Looking after one's health, Walking | | Steps | Armstrong, M, et al (2019) [61] | | 457/231 | | 0.51(0.13,0.88) SMD | | | -1 | | | 0 | | | 0 | | | 0 | | 0 | | M | | | 43% of the included studies have a low risk of bias. |  |  |
| pedometer-based physical activity (PA) promotion / Looking after one's health, Walking and moving | | Steps | Armstrong, M, et al (2019) [61] | | 1485/757 | | 0.53(0.29,0.77) SMD | | | -1 | | | 0 | | | -1 | | | 0 | | 0 | | M | | | 33% of the included studies have a low risk of bias. High heterogeneity (77%). |  |  |
| Fitbit, Jawbone Up24, Combined HR monitor and accelerometer (Actiheart), Wrist-worn accelerometer, FIT Core, Body Media, Fitbug Orb, Polar FA20 accelerometer/ Walking, Weight maintenance functions | | physical activity (total steps, total activity,  the proportion of participants at activity goal) | Tang, MSS, et al (2020) [49] | | 1693/not reported | | 0.449(0.102,0.796) SMD | | | -1 | | | 0 | | | -1 | | | 0 | | 0 | | M | | | None of the included studies have low risk of bias. High heterogeneity (88%). |  |  |
| Accelerometer, Dynaport MoveMonitor, Pedometer, Yamax Digi-walker CW700, ActivPal, ActiGraph, Personal Activity Monitor/ Looking after one's health, Walking | | physical activity (steps per day, energy expenditure, walking time) | Braakhuis, HEM, et al (2019) [67] | | 1432/758 | | 0.34(0.23,0.44) SMD | | | -1 | | | 0 | | | 0 | | | 0 | | 0 | | M | | | 17% of the included studies have low risk of bias. |  |  |
| Fitbit, Jawbone Up24, Combined HR monitor and accelerometer (Actiheart), Wrist-worn accelerometer, FIT Core, Body Media, Fitbug Orb, Polar FA20 accelerometer/ Walking, Weight maintenance functions | | physical activity (total steps, total activity,  the proportion of participants at activity goal) in overweight adults | Tang, MSS, et al (2020) [49] | | 639/not reported | | 0.225(-0.228,0.679) SMD | | | -1 | | | 0 | | | -1 | | | 0 | | 0 | | M | | | None of the included studies have low risk of bias. High heterogeneity (76%). |  |  |
| Fitbit, Jawbone Up24, Combined HR monitor and accelerometer (Actiheart), Wrist-worn accelerometer, FIT Core, Body Media, Fitbug Orb, Polar FA20 accelerometer/ Walking, Weight maintenance functions | | Physical activity (total steps, total activity,  the proportion of participants at activity goal) on healthy adults | Tang, MSS, et al (2020) [49] | | 1054/not reported | | 0.594(0.101,1.08) SMD | | | -1 | | | 0 | | | -1 | | | 0 | | 0 | | M | | | None of the included studies have low risk of bias. High heterogeneity (90%). |  |  |
| Accelerometer, pedometer/ Looking after one's health, Walking | | physical activity (time spent on physical activity,  energy expended on physical activity, step counts,  sedentary time) time measured by questionnaires | Kwan, RYC, et al (2020) [60] | | 2357/1281 | | 53.2(30.18,76.21) MD | | | -1 | | | 0 | | | 0 | | | 0 | | 0 | | M | | | None of the included studies have low risk of bias. |  |  |
| Accelerometer, pedometer/ Looking after one's health, Walking | | physical activity (time spent on physical activity,  energy expended on physical activity, step counts,  sedentary time) time measured by objective wearable devices | Kwan, RYC, et al (2020) [60] | | 851/423 | | 12.95(10.09,15.82) MD | | | -1 | | | 0 | | | 0 | | | 0 | | 0 | | M | | | None of the included studies have low risk of bias. |  |  |
| Pedometer/ Looking after one's health | | Physical activity (steps per day, metabolic equivalents, incidental activity) : combined At completion of intervention (short duration, 1 week) | Freak-Poli, RL, et al (2020) [50] | | 60/31 | | 1.26(0.96,1.66) RM^i^ | | | -1 | | | -1 | | | 0 | | | 0 | | 0 | | M | | | None of the included studies have low risk of bias. Total sample size is 60. |  |  |
| Pedometer/ Looking after one's health | | Physical activity (steps per day, metabolic equivalents, incidental activity): combined At completion of intervention (medium duration, 3 to 6 months) | Freak-Poli, RL, et al (2020) [50] | | 273/152 | | 1.04(0.92,1.17) RM | | | -1 | | | 0 | | | 0 | | | 0 | | 0 | | M | | | None of the included studies have low risk of bias. |  |  |
| Pedometer/ Looking after one's health | | Physical activity (steps per day, metabolic equivalents, incidental activity): combined Follow-up after completion (medium, 3 months) | Freak-Poli, RL, et al (2020) [50] | | 143/96 | | 1.07(0.97,1.18) RM | | | -1 | | | -1 | | | 0 | | | 0 | | 0 | | M | | | None of the included studies have low risk of bias. Total sample size is 143. |  |  |
| Fitbit/ Gait pattern functions, Looking after one's health, Weight maintenance functions | | moderate-to-vigorous physical activity | Ringeval, M. et al (2020) [57] | | 1073/550 | | 6.16(2.8,9.51) MD | | | -1 | | | 0 | | | 0 | | | 0 | | 0 | | M | | | None of the included studies have low risk of bias. |  |  |
| Activity monitor, portable tablet computers with touch screens, Fitbit, Jawbone UP24 wearable device, pedometer, accelerometer/ Looking after one's health,Running,Walking | | moderate-to-vigorous physical activity | Liu, YJ. et al (2020) [59] | | 201/94 | | 0.22(-0.62,1.06) SMD | | | -1 | | | -1 | | | -1 | | | 0 | | 0 | | L | | | None of the included studies have low risk of bias. Optimal information size is 652. High heterogeneity (88%). |  |  |
| Fitbit, Jawbone UP, Polar Active, Misfit Flash, Gruve Solution, LUMOback, BodyMedia Fit, SenseWear, ActiveLink, InBodyBand/ Looking after one's health, Walking | | moderate-to-vigorous physical activity versus control | Lynch, C, et al (2019) [47] | | 512/not reported | | -0.01(-0.15,0.13) SMD | | | -1 | | | -1 | | | 0 | | | 0 | | 0 | | M | | | None of the included studies have low risk of bias.  Optimal information size is 788. |  |  |
| Fitbit, Jawbone UP, Polar Active, Misfit Flash, Gruve Solution, LUMOback, BodyMedia Fit, SenseWear, ActiveLink, InBodyBand/ Looking after one's health, Walking | | moderate-to-vigorous physical activity versus alternative intervention | Lynch, C, et al (2019) [47] | | 833/not reported | | -0.11(-0.2,-0.02) SMD | | | -1 | | | 0 | | | 0 | | | 0 | | 0 | | M | | | None of the included studies have low risk of bias. |  |  |
| Fitbit, Jawbone UP24, Gruve, LumoBack, Polar Active, Fitbug, Pebble+, Fitmeter, Personal Activity Monitor, Withings Pulse/ Walking, Looking after one's health | | Moderate to vigorous physical activity | Brickwood, K, et al (2019) [46] | | 522/260 | | 0.17(0,0.34) SMD | | | -1 | | | -1 | | | 0 | | | 0 | | 0 | | M | | | None of the included studies have low risk of bias. Optimal information size is 790. |  |  |
| Fitbit, Yorbody, AiperMotion/ Looking after one's health | | Moderate to vigorous physical activity | Kirk, MA, et al (2019) [58] | | 1605/829 | | 36.31(18.33,54.29) MD | | | -1 | | | 0 | | | -1 | | | 0 | | -1 | | L | | | None of the included studies have low risk of bias. High heterogeneity (78%).  There is publication bias (the recalculated effect size changed from 36.306, 95%CI: 18.326, 54.287 to 10.165, 95%CI: -8.357, 28.688). |  |  |
| accelerometer, pedometers, Yamax, Fitbit/ Looking after one's health, Walking | | Moderate to vigorous physical activity | Davergne T, et al (2019) [64] | | 117/58 | | 15.83(2.39,29.28) MD | | | -1 | | | -1 | | | 0 | | | 0 | | 0 | | M | | | None of the included studies have low risk of bias.  Total sample size is 117. |  |  |
| Fitbit/ Gait pattern functions, Looking after one's health, Weight maintenance functions | | weight | Ringeval, M. et al (2020) [57] | | 909/480 | | -1.48(-2.81,-0.14) MD | | | -1 | | | 0 | | | 0 | | | 0 | | -1 | | M | | | None of the included studies have low risk of bias.  There is publication bias (the recalculated effect size changed from -1.475 CI: -2.807, -0.142 to -1.269 CI: -2.659, 0.122). |  |  |
| Fitbit, Jawbone Up24, Combined HR monitor and accelerometer (Actiheart), Wrist-worn accelerometer, FIT Core, Body Media, Fitbug Orb, Polar FA20 accelerometer/ Walking,Weight maintenance functions | | weight | Tang, MSS, et al (2020) [49] | | 599/not reported | | 0.133(-0.336,0.603) SMD | | | -1 | | | -1 | | | 0 | | | 0 | | 0 | | M | | | None of the included studies have low risk of bias. Optimal information size is 788. |  |  |
| wristbands, smartwatches/ Weight maintenance functions | | weight | Yen, H, et al (2019) [54] | | 2128/not reported | | -0.594(-0.842,-0.346) HG ^j^ | | | -1 | | | 0 | | | -1 | | | 0 | | 0 | | M | | | None of the included studies have low risk of bias. High heterogeneity (86%). |  |  |
| wristbands, smartwatches/ Weight maintenance functions | | Body-mass index | Yen, H, et al (2019) [54] | | 2023/not reported | | -0.843(-1.29,-0.397) HG | | | -1 | | | 0 | | | -1 | | | 0 | | 0 | | M | | | None of the included studies have low risk of bias. High heterogeneity (94.23%). |  |  |
| wristbands, smartwatches/ Weight maintenance functions | | Waist circumference | Yen, H, et al (2019) [54] | | 528/not reported | | -0.667(-1.309,-0.026) HG | | | -1 | | | 0 | | | -1 | | | 0 | | 0 | | M | | | None of the included studies have low risk of bias. High heterogeneity (87.67%). |  |  |
| Pedometer/ Looking after one's health | | Body-mass index At completion of intervention (medium duration, 3 to 6 months) | Freak-Poli, RL, et al (2020) [50] | | 144/58 | | -1.23(-2.85,0.39) MD | | | -1 | | | -1 | | | 0 | | | 0 | | 0 | | M | | | None of the included studies have low risk of bias. Total sample size is 144. |  |  |
| Fitbit/ Gait pattern functions, Looking after one's health, Weight maintenance functions | | Sedentary behaviors (sitting time, min/day)-objectively measured | Ringeval, M. et al (2020) [57] | | 173/88 | | -10.62(-35.5,14.27) MD | | | -1 | | | -1 | | | 0 | | | 0 | | 0 | | M | | | None of the included studies have low risk of bias. Total sample size is 173. |  |  |
| Fitbit/ Gait pattern functions, Looking after one's health, Weight maintenance functions | | Sedentary behaviors (sitting time, min/day)- self reported | Ringeval, M. et al (2020) [57] | | 528/281 | | -0.11(-0.48,0.26) SMD | | | -1 | | | -1 | | | 0 | | | 0 | | 0 | | M | | | None of the included studies have low risk of bias. Optimal information size is 788. |  |  |
| Fitbit, Jawbone UP24, Gruve, LumoBack, Polar Active, Fitbug, Pebble+, Fitmeter, Personal Activity Monitor, Withings Pulse/ Walking, Looking after one's health | | Sedentary behaviors (sitting time) | Brickwood, K-J, et al (2019) [57] | | 280/139 | | -0.6(-1.39,0.19) SMD | | | -1 | | | 0 | | | -1 | | | 0 | | 0 | | M | | | None of the included studies have low risk of bias. High heterogeneity (84%). |  |  |
| Accelerometer, pedometer/ Looking after one's health, Walking | | Energy expenditure | Kwan, RYC, et al (2020) [60] | | 2123/1308 | | 194.95(87.87,302.04) MD | | | -1 | | | 0 | | | 0 | | | 0 | | 0 | | M | | | None of the included studies have low risk of bias. |  |  |
| Fitbit, Jawbone UP24, Gruve, LumoBack, Polar Active, Fitbug, Pebble+, Fitmeter, Personal Activity Monitor, Withings Pulse/ Walking, Looking after one's health | | Energy expenditure | Brickwood, K, et al (2019) [46] | | 347/174 | | 0.32(0.05,0.58) SMD | | | -1 | | | 0 | | | 0 | | | 0 | | 0 | | M | | | None of the included studies have low risk of bias. |  |  |
| accelerometer, pedometers, Yamax, Fitbit/ Looking after one's health, Walking | | Quality of life | Davergne T, et al (2019) [64] | | 281/150 | | 0.02(-0.22,0.26) SMD | | | -1 | | | -1 | | | 0 | | | 0 | | 0 | | M | | | None of the included studies have low risk of bias.  Optimal information size is 788. |  |  |
| accelerometer, pedometers, Yamax, Fitbit/ Looking after one's health, Walking | | Pain | Davergne T, et al (2019) [64] | | 580/302 | | -0.15(-0.34,0.03) SMD | | | -1 | | | -1 | | | 0 | | | 0 | | 0 | | M | | | None of the included studies have low risk of bias. Optimal information size is 786. |  |  |
| accelerometer-based navigation system/ Involuntary movement reaction functions | | Lower limb mechanical axis outliers | Shigemura, T, et al (2019) [66] | | 1444/684 | | 0.62(0.47,0.82) OR | | | -1 | | | 0 | | | 0 | | | 0 | | 0 | | M | | | None of the included studies have low risk of bias. |  |  |
| accelerometer-based navigation system/ Involuntary movement reaction functions | | coronal femoral component alignment outliers | Shigemura, T, et al (2019) [66] | | 2122/1016 | | 0.31(0.18,0.56) OR | | | -1 | | | 0 | | | 0 | | | 0 | | 0 | | M | | | None of the included studies have low risk of bias. |  |  |
| Accelerometer-based navigation system/ Involuntary movement reaction functions | | Coronal tibial component alignment | Shigemura, T, et al (2019) [66] | | 1640/774 | | 0.66(0.45,0.98) OR | | | -1 | | | 0 | | | 0 | | | 0 | | 0 | | M | | | None of the included studies have low risk of bias. |  |  |
| accelerometer, pedometers, Yamax, Fitbit/ Looking after one's health, Walking | | Functional tests | Davergne T, et al (2019) [64] | | 321/161 | | 0.09(-0.23,0.41) SMD | | | -1 | | | -1 | | | 0 | | | 0 | | 0 | | M | | | None of the included studies have low risk of bias. Optimal information size is 788. |  |  |
| accelerometer, pedometers, Yamax, Fitbit/ Looking after one's health, Walking | | Fatigue | Davergne T, et al (2019) [64] | | 57/31 | | 0.06(-0.46,0.59) SMD | | | -1 | | | -1 | | | 0 | | | 0 | | 0 | | M | | | None of the included studies have low risk of bias. Total sample size is 57. |  |  |
| accelerometer, pedometers, Yamax, Fitbit/ Looking after one's health, Walking | | Disability | Davergne T, et al (2019) [64] | | 339/181 | | 0(-0.22,0.21) SMD | | | -1 | | | -1 | | | 0 | | | 0 | | 0 | | M | | | None of the included studies have low risk of bias. Optimal information size is 788. |  |  |
| Garmin, Pedometer, Fitbit, Accelerometer, Yamax Digiwalker, Gex sensor of vital signs and smartphone/ Aerobic capacity | | Aerobic capacity | Hannan, AL, et al (2019) [48] | | 133/56 | | 1.65(0.64,2.66) MD | | | -1 | | | -1 | | | 0 | | | 0 | | 0 | | M | | | None of the included studies have low risk of bias. Total sample size is 133. |  |  |
| Cardiac monitoring devices | | | | | | | | | | | | | | | | | | | | | | | | | | | | |
| Metronome with a siren, HeartStart-MRx, Zoll AED, Cardio First AngelTM/ Heart functions | | Return of spontaneous circulation (ROSC) | Wang, S. et al (2020) [56] | | 4851/2345 | | 1.42(1.03,1.94) OR | | | 0 | | | 0 | | | 0 | | | 0 | | 0 | | H | | |  |  |  |
| Metronome with a siren, HeartStart-MRx, Zoll AED, Cardio First AngelTM/ Heart functions | | survival-to-discharge | Wang, S. et al (2020) [56] | | 4071/1957 | | 1.27(0.74,2.18) OR | | | -1 | | | 0 | | | -1 | | | 0 | | 0 | | M | | | Almost 71% of the included studies have low risk of bias. High heterogeneity (86%). |  |  |
| Fragmented QRS (fQRS)/ Heart rhythm | | Major arrhythmic events in ICD patients | Kanitsoraphan, C, et al (2019) [70] | | 2231/not reported | | 1.57(0.9,2.74) RR | | | -1 | | | 0 | | | -1 | | | 0 | | 0 | | M | | | None of the included studies have low risk of bias. High heterogeneity (84%). |  |  |
| Fragmented QRS (fQRS)/ Heart rhythm | | Major arrhythmic events in no ICD patients | Kanitsoraphan, C, et al (2019) [70] | | 3632/not reported | | 1.6(1.14,2.25) RR | | | -1 | | | 0 | | | 0 | | | 0 | | 0 | | M | | | None of the included studies have low risk of bias. |  |  |
| Fragmented QRS (fQRS)/ Heart rhythm | | Major arrhythmic events in patients with ejection fraction < 35% | Kanitsoraphan, C, et al (2019) [70] | | 6302/not reported | | 1.41(1.02,1.95) RR | | | -1 | | | 0 | | | 0 | | | 0 | | 0 | | M | | | None of the included studies have low risk of bias. |  |  |
| Fragmented QRS (fQRS)/ Heart rhythm | | Major arrhythmic events in patients with nonspecific ejection fraction | Kanitsoraphan, C, et al (2019) [70] | | 489/not reported | | 7.63(5.15,11.29) RR | | | -1 | | | 0 | | | 0 | | | 0 | | 0 | | M | | | None of the included studies have low risk of bias. |  |  |
| Fragmented QRS (fQRS)/ Heart rhythm | | Major arrhythmic events in patients with ejection fraction < 35 % and nonspecific ejection fraction | Kanitsoraphan, C, et al (2019) [70] | | 6791/not reported | | 1.74(1.09,2.8) RR | | | -1 | | | 0 | | | 0 | | | 0 | | 0 | | M | | | None of the included studies have low risk of bias. |  |  |
| Fragmented QRS (fQRS)/ Heart rhythm | | All-cause mortality in patients with ejection fraction < 35% | Kanitsoraphan, C, et al (2019) [70] | | 6402/not reported | | 1.65(1.2,2.26) RR | | | -1 | | | 0 | | | -1 | | | 0 | | 0 | | M | | | None of the included studies have low risk of bias. High heterogeneity (78%). |  |  |
| Fragmented QRS (fQRS)/ Heart rhythm | | All-cause mortality in patients with nonspecific ejection fraction | Kanitsoraphan, C, et al (2019) [70] | | 489/not reported | | 1.37(0.58,3.22) RR | | | -1 | | | 0 | | | 0 | | | 0 | | 0 | | M | | | None of the included studies have low risk of bias. |  |  |
| Fragmented QRS (fQRS)/ Heart rhythm | | All-cause mortality in patients with ejection fraction < 35% and nonspecific ejection fraction | Kanitsoraphan, C, et al (2019) [70] | | 6891/not reported | | 1.63(1.29,2.19) RR | | | -1 | | | 0 | | | 0 | | | 0 | | 0 | | M | | | None of the included studies have low risk of bias. |  |  |
| Impedance devices/ pressure sensors | | | | | | | | | | | | | | | | | | | | | | | | | | | | |
| Impedance devices/ Heart rhythm | | heart failure related readmission rate | Halawa, A, et al (2019) [51] | | 4164/2255 | | 1.09(0.74,1.6) OR | | | -1 | | | 0 | | | -1 | | | 0 | | 0 | | M | | | 11% of the included studies have low risk of bias. High heterogeneity (79%). |  |  |
| Pressure sensors/ Heart rhythm | | heart failure related readmission rate | Halawa, A, et al (2019) [51] | | 1290/640 | | 1.63(1.1,2.41) OR | | | -1 | | | 0 | | | 0 | | | 0 | | 0 | | M | | | None of the included studies have low risk of bias. |  |  |
| Pressure sensors and Impedance devices (Cardio MEMS, RVP sensor, Chronicle, ICD-OptiVol, InSync Sentry, lung impedance)/ Heart rhythm | | heart failure related readmission rate | Halawa, A, et al (2019) [51] | | 5457/2895 | | 1.25(0.92,1.69) OR | | | -1 | | | 0 | | | -1 | | | 0 | | 0 | | M | | | 7% of the included studies have low risk of bias. High heterogeneity (77%). |  |  |
| Impedance devices/ Heart rhythm | | All-cause mortality | Halawa, A, et al (2019) [51] | | 4164/2255 | | 1.29(0.89,1.86) OR | | | -1 | | | 0 | | | 0 | | | 0 | | 0 | | M | | | 11% of the included studies have low risk of bias. |  |  |
| Pressure sensors/ Heart rhythm | | All-cause mortality | Halawa, A, et al (2019) [51] | | 1292/641 | | 1.04(0.62,1.74) OR | | | -1 | | | -1 | | | 0 | | | 0 | | 0 | | M | | | None of the included studies have low risk of bias. Optimal information size is 2008. |  |  |
| Pressure sensors and Impedance device (Cardio MEMS, RVP sensor, Chronicle, ICD-OptiVol, InSync Sentry, lung impedance)/ Heart rhythm | | All-cause mortality | Halawa, A, et al (2019) [51] | | 5456/2896 | | 1.21(0.91,1.61) OR | | | -1 | | | 0 | | | 0 | | | 0 | | 0 | | M | | | 8% of the included studies have low risk of bias. |  |  |
| Impedance devices/ Heart rhythm | | Combined HF^k^ related readmission and all-cause death | Halawa, A, et al (2019) [51] | | 4164/2255 | | 1.05(0.71,1.55) OR | | | -1 | | | 0 | | | -1 | | | 0 | | 0 | | M | | | 12.5% of the included studies have low risk of bias. High heterogeneity (84%). |  |  |
| Pressure sensors/ Heart rhythm | | Combined HF related readmission and all-cause death | Halawa, A, et al (2019) [51] | | 1290/640 | | 1.58(1.07,2.34) OR | | | -1 | | | 0 | | | 0 | | | 0 | | 0 | | M | | | None of the included studies have low risk of bias. |  |  |
| Pressure sensors and Impedance devices (Cardio MEMS, RVP sensor, Chronicle, ICD-OptiVol, InSync Sentry, lung impedance)/ Heart rhythm | | Combined HF related readmission and all-cause death | Halawa, A, et al (2019) [51] | | 5457/2898 | | 1.21(0.89,1.64) OR | | | -1 | | | 0 | | | -1 | | | 0 | | 0 | | M | | | 8% of the included studies have low risk of bias. High heterogeneity (81%) |  |  |

^a^ ICD: Implantable cardiac defibrillator, ^b^ OR: odds ratio, ^c^ RR: Risk ratio, ^d^ CF-LVAD: continuous-flow left ventricular assist devices, ^e^ HR: Hazard ratio, ^f^ CRT: Cardiac resynchronization therapy, ^g^ MD: mean difference, ^h^ SMD: standardized mean difference, ^i^ RM: ratio of means, ^j^ HG: Hedges’ g, ^k^ HF: heart failure, ^l^ M: moderate quality of evidence, ^m^ H: high quality of evidence, ^n^ L: low quality of evidence.
